# High-efficiency c-Myc-mediated induction of functional hepatoblasts from the human umbilical cord mesenchymal stem cells

**DOI:** 10.1186/s13287-021-02419-1

**Published:** 2021-07-02

**Authors:** Jie Deng, Kai Luo, Pengchao Xu, Qingyuan Jiang, Yuan Wang, Yunqi Yao, Xiaolei Chen, Fuyi Cheng, Dan Xie, Hongxin Deng

**Affiliations:** 1 State Key Laboratory of Biotherapy and Cancer Center/Collaborative Innovation Center of Biotherapy, West China Hospital, Sichuan University, Chengdu, 610041 People’s Republic of China; 2 Center for Cell Lineage and Atlas (CCLA), Bioland Laboratory (Guangzhou Regenerative Medicine and Health Guangdong Laboratory), Guangzhou, 510005 China; 3 National Frontier Center of Disease Molecular Network, West China Hospital, Sichuan University, Chengdu, 610041 China; 4 Department of Obstetrics, Sichuan Provincial Hospital for Women and Children, Chengdu, China

**Keywords:** Hepatoblast-like cells, Human umbilical cord mesenchymal stem cells, c-Myc, Direct reprogramming

## Abstract

**Background:**

Direct reprogramming of human fibroblasts to hepatocyte-like cells was proposed to generate large-scale functional hepatocytes demanded by liver tissue engineering. However, the difficulty in obtaining large quantities of human fibroblasts greatly restricted the extensive implementation of this approach. Meanwhile, human umbilical cord mesenchymal stem cells (HUMSCs) are the preferred cell source for HLCs with the advantages of limited ethical concerns, easy accessibility, and propagation in vitro. However, no direct reprogramming protocol for converting HUMSCs to hepatoblast-like cells (HLCs) has been reported.

**Methods:**

HLCs were successfully generated from HUMSCs by forced expression of FOXA3, HNF1A, and HNF4A (collectively as 3TFs) and c-Myc. In vitro and in vivo functional experiments were conducted to demonstrate the hepatic phenotype, characterization, and function of HUMSC-derived HLCs (HUMSC-iHeps). ChIP-seq and RNA-seq were integrated to reveal the potential molecular mechanisms underlying c-Myc-mediated reprogramming.

**Results:**

We showed that c-Myc greatly improved the trans-differentiation efficiency for HLCs from HUMSCs, which remained highly efficient in reprogramming fibroblasts into HLCs, suggesting c-Myc could promote direct reprogramming and its potentially widespread applicability for generating large amounts of HLCs in vitro. Mice transplantation experiments further confirmed the therapeutic potential of HUMSC-iHeps by liver function restoration and survival prolongation. Besides, in vivo safety assessment demonstrated the low risk of the tumorigenic potential of HUMSC-iHeps. We found that c-Myc functioned predominantly at an early phase of reprogramming, and we further unraveled the regulatory network altered by c-Myc.

**Conclusions:**

c-Myc enhanced reprogramming efficiency of HLCs from HUMSCs. A large scale of functional HLCs generated more conveniently from HUMSCs could benefit biomedical studies and applications of liver diseases.

**Supplementary Information:**

The online version contains supplementary material available at 10.1186/s13287-021-02419-1.

## Background

Liver transplantation is currently the only curative treatment for liver diseases, which caused approximately millions of deaths each year [1]. Primary human hepatocyte (PHH) transplantation has been evaluated in clinics as an alternative to organ transplantation [2]. Despite progress made in PHHs [3–5], the inability to expand in vitro has been a bottleneck for clinical applications of PHHs [2, 6, 7]. In addition, the demand for functional hepatocytes far exceeds the supply of living donors [2]. Therefore, the generation of a large number of functional hepatoblast-like cells (HLCs) is of significance to clinical applications [8].

Currently, HLCs could be obtained mainly by three methods, induced pluripotent stem cell (iPSCs), direct reprogramming, and differentiation of pluripotent stem cells [9–11]. Major weaknesses of iPSCs include low induction efficiency and time-consuming generation process [12]. Direct reprogramming as an alternative to iPSCs, enables rapid derivation of hepatocyte-like cells with a simplified process [13, 14]. However, most direct reprogramming approaches used somatic cells, for example, fibroblasts as source cells, which have disadvantages of low proliferation capacity and invasive sampling. Human umbilical cord mesenchymal stem cells (HUMSCs) derived from the neonatal umbilical cord are easily accessible and expandable with limited ethical concerns [15, 16]. Moreover, banks of HUMSCs are being set up in countries all over the world. If the direct programming protocol could apply on HUMSCs, large quantities of HLCs would be obtained in vitro with no invasive surgery for patients.

Huang et al. firstly reported the direct reprogramming of human fibroblasts to HLCs by lentiviral expression of FOXA3, HNF1A, and HNF4A (collectively as 3TFs) [13]. In this study, we explored the possibility of generating HLCs from HUMSCs by 3TFs, while low induction efficiency was observed. Referring to researches on iPSCs, c-Myc, which can greatly improve the efficiency of reprogramming during iPSC induction [17], were added together with 3TFs to improve efficiency. Herein, we explored and confirmed that c-Myc could significantly promote the induction efficiency of HUMSCs into HLCs. The HLCs derived from HUMSCs (HUMSC-iHeps) with 3TFs and c-Myc, harbored typical morphological characteristics and hallmark functions of hepatocytes. Besides, we demonstrated that HUMSC-iHeps presented high proliferation activity in vitro and therapeutic effects on acute liver failure in mice. We further uncovered the molecular mechanism underlying the enhanced efficiency of c-Myc by integrating RNA-seq and ChIP-seq. Intriguingly, the promoting effect of c-Myc retained in direct reprogramming of fibroblasts to HLCs. These results may inspire further exploration on generating HLCs with great clinical significance for liver diseases in the future.

## Methods

### Isolation, culture, and expansion of HUMSCs

The neonate umbilical cord was obtained from a health donor in the Sichuan Maternal and Child Health Hospital with the consent of the donor according to procedures approved by the Medical Ethics Committee, Sichuan University (K2018109-1). The neonate umbilical cord was washed with Hank’s balanced salt solution (HBSS, Gibco) three times and removed the veins and arteries. The residual umbilical cord tissue was minced into small pieces and digested in 0.1% collagenase type I (25ml for each 10 mL of tissue, Gibco) at 37°C for 0.5 h with gentle shaking at 120 rpm. Then, an equal volume of cold low glucose DMEM (DMEM-LG, Gibco) was added to terminate digestion. For further disintegration of tissue aggregates, the sample was pipetted up and down several times. The cell suspension was filtered through sterile gauze for the removal of the solid aggregates. The sample was subsequently centrifuged at 1500 rpm for 3 min at 4°C and completed the separation of the stromal cells. The cell pellet was resuspended in complete medium (DMEM-LG with 10% FBS and 1% antibiotic antimycotic solution) in 10-cm culture dishes and maintained in an incubator supplied with humidified atmosphere of 5% CO_2_ at 37°C. After 1 day, non-adherent cells were removed by two to three washes with HBBS and adherent cells further cultured in complete medium. The medium was changed every 2 days until the monolayer of adherent cells reached 80–90% confluence. Cell passaging was performed using 0.25% trypsin solution (Sigma). The number of recovered cells evaluated with the use of hemocytometer and cellular viability was quantified by the Trypan Blue exclusion test. Approximately, 3×10^5^ cells were used to inoculate a 10-cm culture dish and continue to be incubated.

### Human skin fibroblasts and PHHs

Human skin fibroblasts (HSFs) were obtained from Professor Yang Yang’s laboratory. HSFs were seeded on a collagen-I-coated dish and infected with lentiviruses carrying the indicated genes. Primary human hepatocytes (PHHs) were freshly isolated from liver hemangioma with informed consents from the patients. Institutional ethical committees of the Western China Hospital approved the collection and use of these human samples.

### Lentivirus production

Modified pCDH plasmids carrying candidate genes were introduced into HEK293T cells together with packaging plasmid psPAX2 (Addgene) and envelope plasmid pMD2.G (Addgene) to produce viruses.

### In vitro differentiation of HUMSCs and HSFs into HLCs

At passages 3 to 7, the cells were plated on collagen type I-coated dishes at a concentration of 2.0×10^4^ cells/cm^2^. When cells adherence, cells were infected with lentivirus of FOXA3, HNF1α, HNF4α, and c-Myc or not. After 12 h, the cells were transferred to fresh media. Hepatic induction was carried out 2 days after infection. First, the cells were treated for 4 days of reprogramming media. Second, the culture medium was replaced with hepatogenic medium every 12 days. Finally, the epithelioid clones were picked and cultured in hepatogenic medium. Cell passaging was performed using 0.25% trypsin solution.

### Immunofluorescence

Firstly, the cells were fixed with 4% paraformaldehyde for 15 min at room temperature and then incubated with PBS containing 0.2% Triton X-100 (Sigma) for 15 min. Cells were then washed three times with PBS. After being blocked by 5% BSA in PBS for 60 min at room temperature, cells were incubated with primary antibodies at 4°C overnight, washed three times with PBS, and then incubated with appropriate fluorescence-conjugated secondary antibody for 60 min at 37°C in the dark. Nuclei were stained with DAPI (Sigma). Primary and secondary antibodies were diluted in PBS containing 3% BSA.

### Real-time quantitative PCR

Total RNA was isolated from cells using the Trizol Reagent (Invitrogen). The sequences of both the forward and reverse primers are listed in Table S1. In parallel, we analyzed the mRNA concentration of the human housekeeping β-actin as an internal control for normalization. The real-time monitoring of the PCR reaction, the precise quantification of the products in the exponential phase of the amplification, and the melting curve analysis were performed with the Bio-Rad CFX Manager software, following recommended instructions of the manufacturer.

### Assays for PAS, ac-LDL, and ICG

Cells were stained by the Periodic-Acid-Schiff (PAS, Sigma-Aldrich) and DiI-LDL (Invitrogen). For indocyanine green (ICG, Sigma-Aldrich) uptake assay, cells were changed medium with 1 mg/ml ICG and incubated at 37°C for 1 h, followed by washing with PBS three times.

### Human albumin and a-1-antitrypsin ELISA

Human albumin and a-1 antitrypsin were measured by the human albumin ELISA quantitation set (Bethyl Laboratory) and the human a-1-antitrypsin ELISA kit (Bethyl Laboratory).

### Transplantation of HUMSC-iHeps cells into CCl_4_/retrorsine-induced model mice

All mice experiments were performed in accordance with institutional regulations. Female BALB/c nude mice (approximately 7 weeks old) were pretreated with 70 mg/kg retrorsine for at the 30th and 14th day before cell transplantation. One day before transplantation, mice were treated with 0.5mL/kg CCl_4_ and 50μL Anti-asialo GM1 (Wako Pure Chemical Industries). 7.5 μg/ml of FK506 was added to the drinking water of mice until the end of the experiment. HUMSC-iHeps (1×10^7^ cells per mouse) were intrasplenically transplanted into retrorsine-treated mice after the CCl_4_-injected. After cell transplantation, each mouse was injected 50-mg anti-asialo GM1 antibody every week to decrease natural killer cells. Body weight was monitored twice a week post transplantation. Surviving recipient mice were sacrificed to collect blood and liver samples 9 weeks after transplantation.

### HUMSC-iHeps cell transplantation to concanavalin-A-induced acute liver failure mice

BALB/c mice were injected with concanavalin A (Con A) (Sigma-Aldrich) at a dose of 37 mg/kg through tail vein to trigger acute liver failure. Metrigel-mixed PBS (200μL PBS+200μL Matrigel), 2×10^6^ metrigel-encapsulated PHHs, and 2×10^6^ metrigel-encapsulated HUMSC-iHeps (200μL cells+200μL Matrigel) were injected intraperitoneally into acute liver failure mice. Blood samples were collected from surviving mice for 24-h interval. Liver samples were collected after the surviving animals were sacrificed.

### CYP induction and metabolism assay

To measure expression of Cytochrome P450 (CYP) enzyme, three cell types (HUMSC-iHeps, HSF-iHeps, and PHHs) were cultured in HIM for 48 h and then change to HIM media supplemented with or without 3-methylcholanthrene (25μM), rifampicin (25μM), and sodium phenobarbital (2mM) for additional 48 h. CYP1A2 was induced by 3-methylcholanthrene. CYP2A6, CYP2C8, and CYP2C9 were induced by rifampicin. CYP2B6 and CYP3A4 were induced by phenobarbital. The total RNA was extracted to measure the induction of CYP enzymes responding to chemical inducers by qPCR. The total RNA from cells without inducer treatment was used to measure the expression of CYP450 genes.

### RNA-Seq analysis

The total RNA was extracted from HUMSCs, HSFs, fresh primary human hepatocytes, HUMSC-iHep cells at passage 4, and inducing HUMSC-iHep cells at 6th day with/without c-Myc infected with miRNeasy mini kit (217004,Qiagen) and poly(A)+ mRNAs was purified with NEBNext Poly(A) mRNA Magnetic Isolution Module. Sequencing libraries were generated using NEBNext Ultra RNA Library Prep Kit (NEB, USA) and sequenced to 150bp paired-end reads on Illumina Hiseq system. Clean RNA-seq reads were aligned to the hg38 genome assembly using Hisat2 (version 2.0.4), and gene expression was quantified by StringTie (version 1.3.0). Gene count matrix was generated following the instructions on StringTie manual using a Python script (prepDE.py), and differential expression genes were further identified by DESeq2 (version 1.26.0). Gene Set Enrichment Analysis (GSEA) was performed with GSEA (version 4.0.0) to identify the pathways that were significantly enriched.

### ChIP-seq analysis

ChIP was performed according to the manufacturer’s instructions (Cell Signaling Technology, # 9003S). Briefly, the chromatin/DNA proteins complexes were prepared from the 6th day inducing HUMSC-iHep cells with/without c-Myc infected. Chemical cross-linking of DNA-proteins was carried out using 1% formaldehyde for 10 min at room temperature. The cross-linking was quenched by addition of glycine (0.125 M) for 5 min at room temperature and followed by two washes with ice-cold PBS. Cells were scraped into PBS containing 1mM PMSF. The cell suspension was centrifuged, and the pellet was mixed by inverting the tube every 3 min in buffer A + DTT+ PIC+ PMSF, incubated on ice for 10 min. The pellet (nuclei) was dissolved in 1.0 mL buffer B +DTT + 5 μL of micrococcal nuclease and incubated for 20 min at 37°C with frequent mixing to digest DNA to length of approximately150–900 bp. The lysate was incubated with appropriate chip-grade c-Myc antibody (CST) to immunoprecipitate chromatin overnight at 4°C with rotation and followed by ChIP-grade protein G magnetic beads and incubation for 2 h at 4°C with rotation. The magnetic beads were washed using buffers supplied with the kit. The eluted DNA was purified and analyzed by PCR to determine the binding of c-Myc to chromatin. PCR primers were designed by using the criteria described in the kit. ChIP DNA libraries were sequenced to 150bp pair-end reads on Illumina Hiseq system. Reads were aligned to hg38 genome assembly using Bowtie (version 1.2.1). PCR duplicate reads were removed by Picard (version 2.19.0). MACS2 (version 2.1.0) were used to detect c-Myc binding sites.

### Statistics

Data were analyzed using unpaired Student’s t test. For survival analysis, the Mantel-Cox log rank test was applied. Statistic calculation was performed using Statistical Program for Social Sciences software (SPSS, IBM). For all statistics, data from at least three independent samples were used. Differences were considered statistically significant for *p* values < 0.05.

## Results

### c-Myc enhanced reprogramming efficiency of HUMSCs to HLCs

Direct reprogramming of human fibroblasts into hepatocytes was firstly reported to be successfully inducted by FOXA3, HNF1A, and HNF4A [13]. Considering the limited proliferative ability and difficulty in acquiring large numbers of human fibroblasts, we wished to generate functional HLCs from HUMSCs, because of their easy accessibility and limited ethical concerns.

**Fig. 1:**
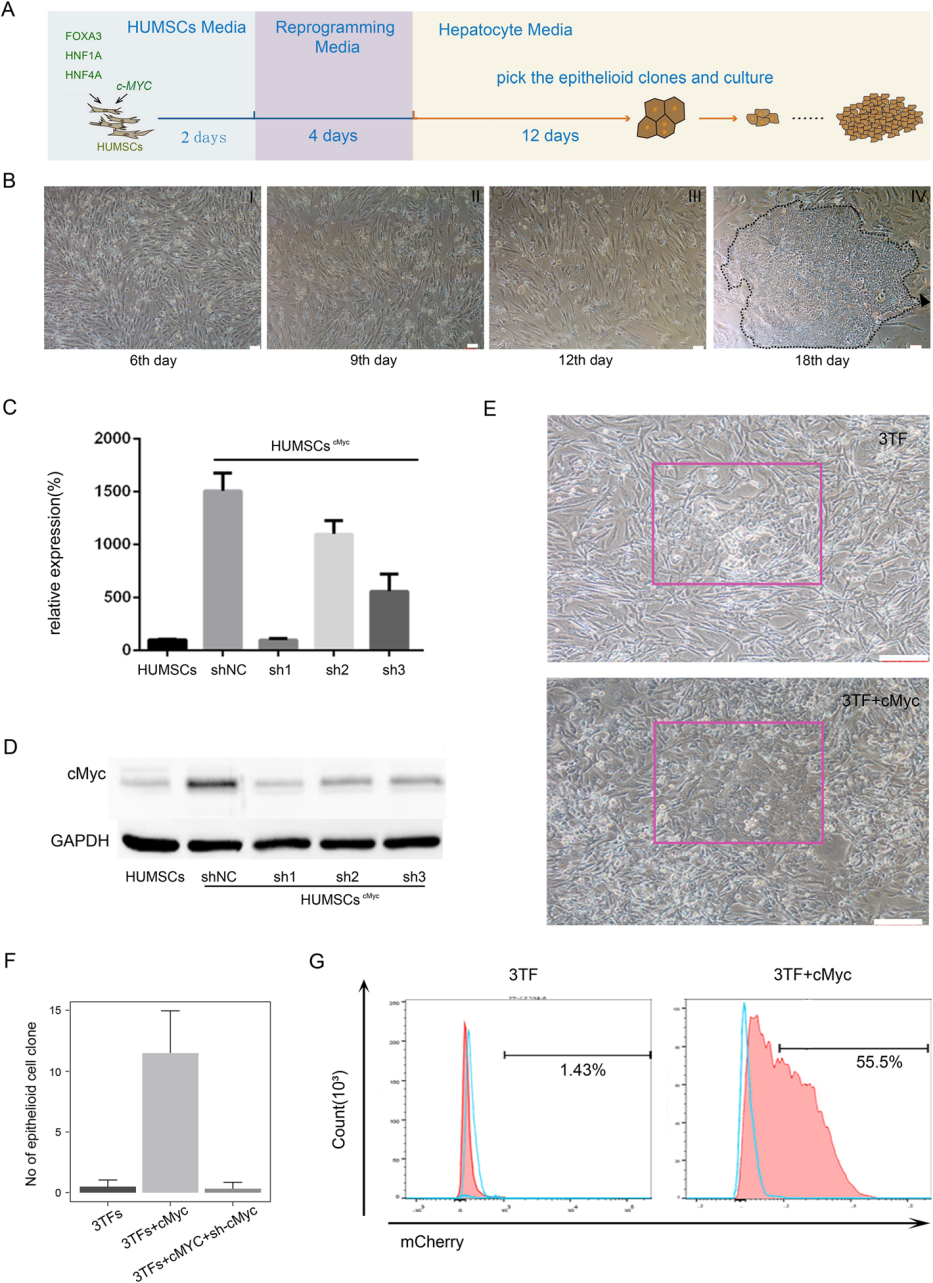
Induction of HUMSCs into HLCs. **A** Schematic diagram of the reprogramming process. **B** Sequential changes in cell morphology during the induction process. (I) some apoptotic cells appeared on the 6th day. (II) Short-spindle cells showed on the 9th day. (III) Cells with irregular shape showed on the 12th day. (IV) Epithelial cell clones appeared as shown in the dotted line on the 18th day. **C**, **D** Interference efficiency of shRNAs. **C** c-Myc expression in HUMSCs with/without shRNA interference detected by qPCR. **D** The knockout efficiency of sh1, sh2, and sh3 on c-Myc detected by Western blot. **E** Images shown epithelial cell clones in two induced groups of HUMSCs: 3TFs and 3TFs+c-Myc. Scale: 100μm. **F** Number of epithelial cell clones in three induction groups: 3TFs, 3TFs+c-Myc, and 3TFs+c-Myc+ sh-c-Myc. *P <0.05, t test. The data presented as mean±SD. U.D. stands for undetected. **G** Flow cytometry analysis of HUMSC-iHeps infected with TTR-mCherry lentivirus

We first isolated the umbilical cord mesenchymal stem cells (HUMSCs) and demonstrated their identity and characteristics (Figure S1). Lentiviruses carrying FOXA3, HNF1A, and HNF4A were introduced into HUMSCs. However, little epithelial-like cell clones were attained and all cells died due to low proliferative capacity and low passaging capability (Table S2), suggesting that an improved protocol is required for generating HLCs from HUMSCs. Based on findings in iPSCs [17], we considered adding c-Myc to promote the induction efficiency. The induction process was depicted in Fig. 1A. Many cells died after 6 days of lentiviruses infection (Fig. 1B, I), and some short spindle cells could be observed on 9 days post infection (Fig. 1B, II), gradually irregular shaped cells were shown on the 12th day (Fig. 1B, III). On the 18th day, epithelioid clones (shown in phantom circles) appeared (Fig. 1B, IV).

To examine whether c-Myc could increase the reprogramming efficiency into HLCs, we analyzed the induction efficiency of the three HUMSCs induction groups: (1) 3TFs group, (2) 3TFs+c-Myc group, and (3) 3TFs+c-Myc+sh-c-Myc group. We designed three sh-c-Myc sequences and chose the sequence with the best downregulated efficiency to ensure target specificity (Fig. 1C-D, Table S3). As stated above, we obtained a few epithelial-like cell clones in 3TFs group, whereas the 3TFs+c-Myc group gave rise to 7–17 colonies of epithelial-like cell clones (Fig. 1E). We originally plated 2*10^5 HUMSCs per well, and got 0–1 colonies (*n* = 6; 0, 0, 0, 1, 1, 1 colonies, respectively) in the 3TFs group, while got 7–17 colonies (*n* = 6; 7, 9, 11, 12, 13, 17 colonies, respectively) in the 3TFs+cMyc group, i.e., the differentiated rate of HUMSCs into HLCs of the 3TFs+cMyc group ranged from 3.5e−05 to 8.5e−05, higher than that in the 3TFs group. What is more, induced cells from the former group exhibited low proliferative capacity, low passaging capability, and all cells died. Nevertheless, cell clones derived from the latter group could be cultured as hepatocyte-like cells (as shown in the red box, Fig. 1E). Once c-Myc was interfered by shRNA, and no epithelial-like cell clones could be observed (Fig. 1F). Crystal violet staining was not appropriate to show cell clone number, for the reason that the epithelial-like cell clones were flattened and spread out, and did not aggregate into tightly stacked cell clusters (as shown in the red box, Fig. 1E). Instead, to quantify the reprogramming efficiency, cells were directly passaged and then infected with TTR-mCherry lentivirus which composed of a TTR promoter linked with the mCherry gene. As a result, the ratio of red fluorescent in the 3TFs+c-Myc group was 55.5%, ~39 fold larger than that in 3TFs alone group (1.43%), indicating that c-Myc increased the number of HLCs and enhanced the efficiency of direct reprogramming (Fig. 1G). Epithelioid clones shown on 18 days after 3TFs+c-Myc induction (Fig. 1B, IV) in the dotted circle were picked and cultured to get hepatocyte-like cells (HUMSC-iHeps).

### HUMSC-iHeps possessed characteristics and functions specific for hepatoblasts

**Fig. 2:**
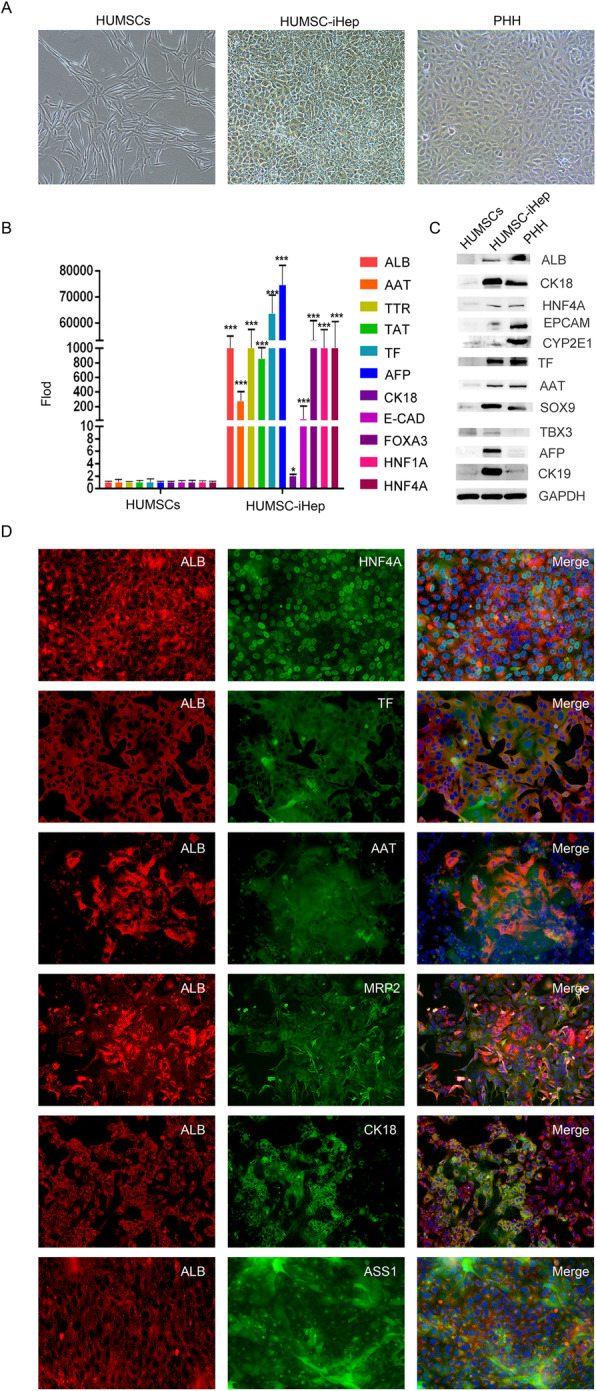
HUMSC-iHeps possessed characteristics of hepatocytes. **A** Images of HUMSCs, HUMSC-iHeps, and PHHs. **B** Hepatic gene expression of HUMSCs and HUMSC-iHeps was measured by RT-PCR. Data was normalized to HUMSCs. **C** Hepatic gene expression of HUMSCs, HUMSC-iHeps, and PHHs was measured by western blot. **D** Co-expression of the mature hepatic marker ALB, and other hepatic markers HNF4A, TF, AAT, MRP2, CK18, and ASS1 were determined by immunofluorescence staining in HUMSC-iHeps

Different from HUMSCs, pavement HUMSC-iHeps formed a mosaic shape similar to primary liver cells (Fig. 2A). Compared with uninduced HUMSCs, hepatocyte-specific genes, such as ALB, AAT, TTR, TF, AFP, FOXA3, HNF1A, and HNF4A, were significantly upregulated in both mRNA and protein levels in HUMSC-iHeps (Fig. 2B-C). In addition, immunofluorescence staining further confirmed that HUMSC-iHeps expressed hepatocyte-specific markers, for example, ALB, HNF4A, AAT, TF, CK18, MRP2, and ASS1 (Fig. 2D). Taken together, the pavement morphology and marker gene expression of HUMSC-iHeps suggested that they harbored typical characteristics of *hepatoblasts.*

**Fig. 3:**
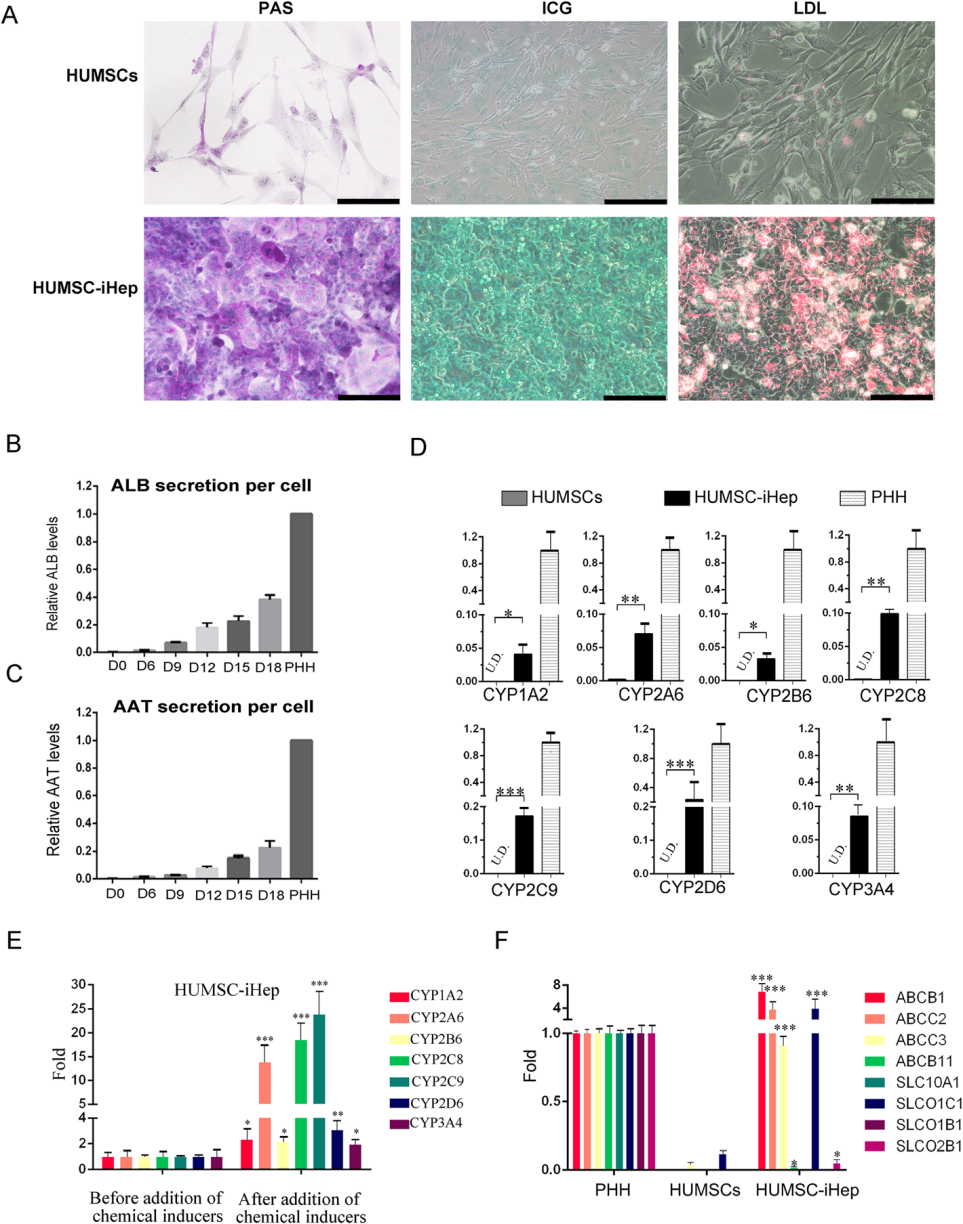
HUMSC-iHep possessed hallmark functions of hepatocytes. **A** Basic liver function analysis of HUMSC-iHeps, including PAS staining and uptake of ICG and LDL. Scale bar: 100 μm. **B**, **C** Secretion of ALB (**B**) and AAT (**C**) increased during the induction process as measured by ELISA. **D** The mRNA levels of CYP genes in HUMSCs, HUMSC-iHeps, and PHHs before inducer treatment were determined by qPCR. Data normalized to the levels in PHH. *P < 0.05, t test. Data are represented as the mean ± SD. UD undetectable. **E** The mRNA levels of the induced CYP enzymes were measured by qPCR. CYP1A1 and CYP1A2 were induced by 3-methylcholanthrene. CYP2A1 and CYP3A1 were induced by phenobarbital. CYP2E1 was induced by acetone. Fold induction in HUMSC-iHeps and PHHs was normalized to expression in corresponding cells without inducer treatment. *P < 0.05, t test. Data are represented as the mean ± SD. **F** Expression of drug transporter genes in HUMSC-iHeps determined by qPCR. Data are normalized to PHHs. *P < 0.05, t test. Data are represented as the mean ± SD

Moreover, HUMSC-iHeps displayed hallmark functions of mature hepatocytes, including glycogen storage, low-density lipoprotein (LDL) uptake, and indocyanine green (ICG) uptake (Fig. 3A). Besides, we collected culture media at different time points for ELISA assay and found the concentration of human ALB and AAT secreted by these cells was gradually increasing (Fig. 3B, C). These results further confirmed that the HUMSC-iHeps possessed typical functions of hepatoblasts.

We next analyzed whether HUMSC-iHeps were responsive to CYP inducers. Before adding inducer, HUMSC-iHeps expressed several CYP450 enzyme genes, including CYP1A2 (an AHR target gene), CYP2A6 (a CAR target gene), CYP2B6, CYP2C8, CYP2C9 (target genes of CAR and PXR), CYP2D6, and CYP3A4, but their expression level was not as high as that observed in PHHs (Fig. 3D). After treatment of chemical inducer 3-methylcholanthrene, phenobarbital, and rifampicin treatment, significantly elevated mRNA expression of CYP450 enzymes were detected (Fig. 3E). While CYP2A6, CYP2C8, and CYP2C9 showed the highest expression increase, the other three genes (CYP1A2, CYP2B6, CYP3A4) showed the least increase. It revealed that HUMSC-iHeps partially developed function of drug metabolism (Fig. 3E).

Membrane transporter-mediated biliary excretion is another hallmark function of hepatocytes for clearance of xenobiotics. Much high expression levels of key transporter genes, such as ABCB1, ABCC2, ABCC3, and SLCO1C1, were observed in HUMSC-iHeps with expression level close to or even exceed that in PHHs (Fig. 3F). All these results above strongly confirmed that the HUMSC-iHeps possessed characteristics and hallmark functions of hepatocytes.

### c-Myc promoted direct reprogramming of fibroblasts into HLCs

In addition to mesenchymal stem cells, we hypothesized that c-Myc could also promote 3TFs reprogramming in other cell types, such as human skin fibroblasts (HSFs). So, we also added c-Myc to 3TFs-induced human skin fibroblasts to repeat the experiments which performed in HUMSCs. And sure enough, the HSFs could also be reprogrammed into HLCs and similar results were achieved. The HSFs-derived hepatocyte-like cells (HSF-iHeps) also possessed the characteristics (Figure S2) as well as hallmark functions of hepatocytes (Figure S3) in vitro. These results demonstrated that c-Myc cannot only effectively improve the efficiency of 3TFs reprogramming in mesenchymal stem cells but also in somatic cells, indicating that this reprogramming process did not rely on the differentiation potential of mesenchymal stem cells.

### HUMSC-iHeps exhibited an expandable bi-phenotypic status

**Fig. 4:**
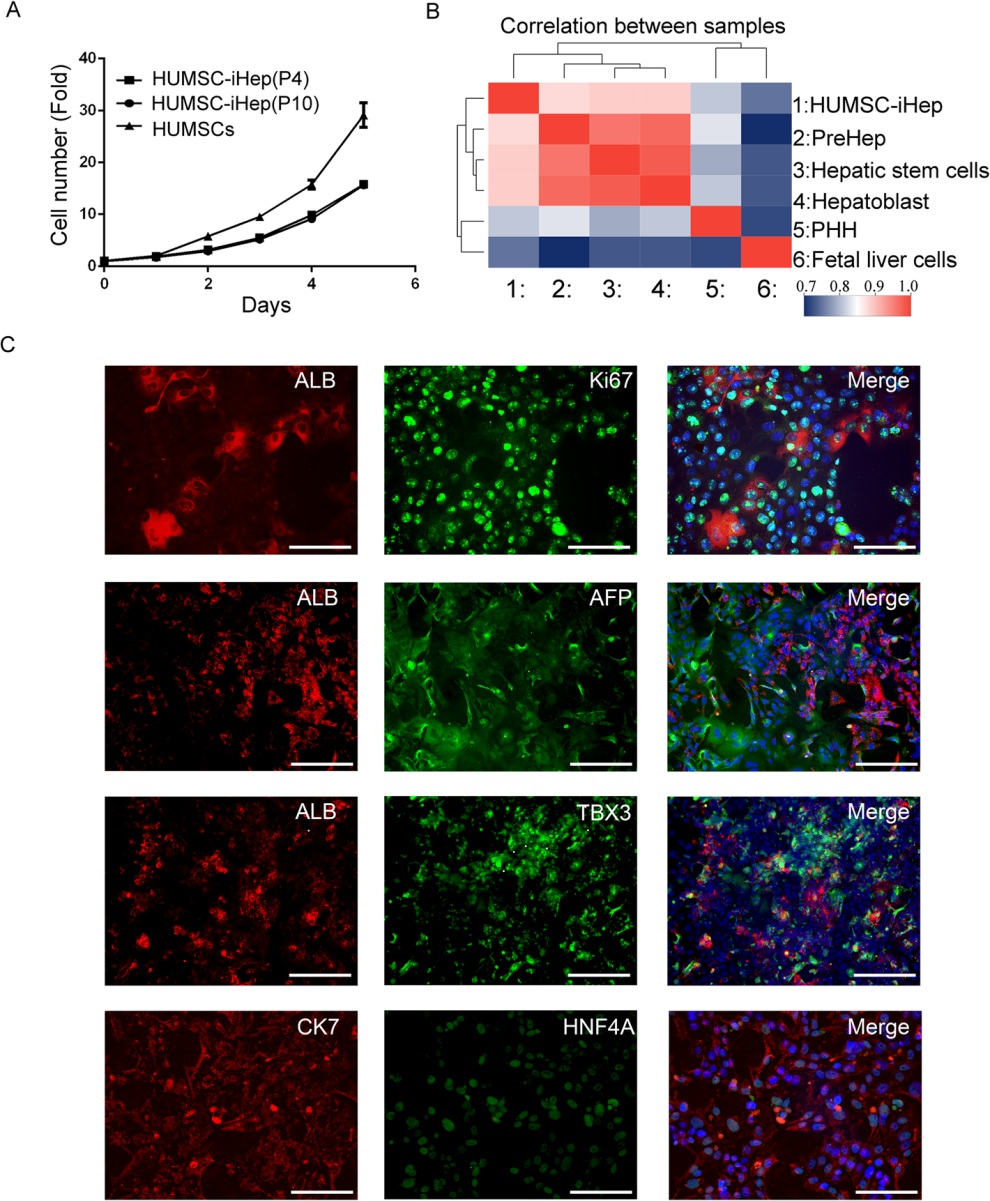
HUMSC-iHeps displayed characteristics of both mature hepatocytes and progenitor cells. **A** Proliferative capacity of HUMSC-iHeps. **B** Transcriptome correlation analysis of HUMSCs, PHHs, HUMSC-iHep, HSF-iHep, hepatoblast, and fetal liver cells. **C** The co-expression of ALB, HNF4A, and AFP, TBX3, and CK7 in HUMSC-iHeps was determined by immunofluorescence staining. Scale: 100μm

The cell doubling time was about 2.5 days for both P4 and P10 of HUMSC-iHeps (Fig. 4A), suggesting powerful cell proliferation capacity of HUMSC-iHeps.

Correlation analysis of transcriptomes of HUMSC-iHeps and other liver cells, including hepatoblast, hepatic stem cells, PreHep, fetal liver cells, and PHHs, unraveled relatively high correlation, ranging from 0.75 to 0.89. And correlation between HUMSC-iHeps and PHHs was 0.81, confirming hepatic-specific transcriptome of HUMSC-iHeps. Notably, compared with PHHs, HUMSC-iHeps was much closer to liver progenitor cells (hepatoblast, hepatic stem cells, PreHep) as hierarchical clustering showed (Fig. 4B).

Importantly, the expression of hepatic-related transcriptional factors was upregulated, and the expression of fibroblasts and mesenchyme stem cell marker genes was repressed in HUMSC-iHeps (Figure S4A-B). Moreover, genes involved in glucose metabolism, lipid cholesterol metabolism, fatty acid metabolism, and drug metabolism showed higher expression in HUMSC-iHeps than that in other cells (Figure S4B). Gene Set Enrichment Analysis (GSEA) also showed that pathways enriched in PHHs were also enriched in HUMSC-iHeps, including lipid metabolism, amino acid metabolism, and glucose metabolism (Figure S5), unraveling the similarity between HUMSC-iHeps and PHHs.

Western blot experiments also confirmed that HUMSC-iHeps and HSF-iHeps expressed markers of hepatic progenitor cells along with markers of mature hepatocytes (Fig. 2C and Figure S2B). Immunofluorescence staining (Fig. 4C) showed that the Ki67 highly expressed in HUMSC-iHeps, and in some cells co-expressed with ALB, indicating that the HUMSC-iHeps was in proliferative state but still retained the expression of some mature hepatic genes, thus implying that HUMSC-iHeps were heterogeneous population consisting of mature and immature hepatic cells. Mature hepatocyte marker (ALB, HNF4A) and hepatic progenitor markers (TBX3, AFP, CK7) co-expressed in HUMSC-iHeps, but their expression was spatial complementarity, suggesting that HUMSC-iHeps harbored the expression pattern of both hepatic progenitor cells and mature hepatocytes, and the maturity of HUMSC-iHeps was uneven (Fig. 4C).

In addition, mature bile ducts associated genes, such as cystic fibrosis transmembrane conductance regulator (CFTR), secretin receptor (SCTR), somatostatin receptor 2 (SSTR2), and aquaporin-1 (AQP1), were all not expressed in HUMSC-iHeps (Figure S4C), indicating that HUMSC-iHeps was not differentiated into bile duct epithelial cells.

The above results confirmed that HUMSC-iHeps harbored the characteristics of liver progenitor cells while maintaining basic functions of liver cells, implying that these cells had enormous potential for in vitro amplification and was conducive to large-scale application.

### Transplantation of HUMSC-iHeps improve acute liver failure

**Fig. 5:**
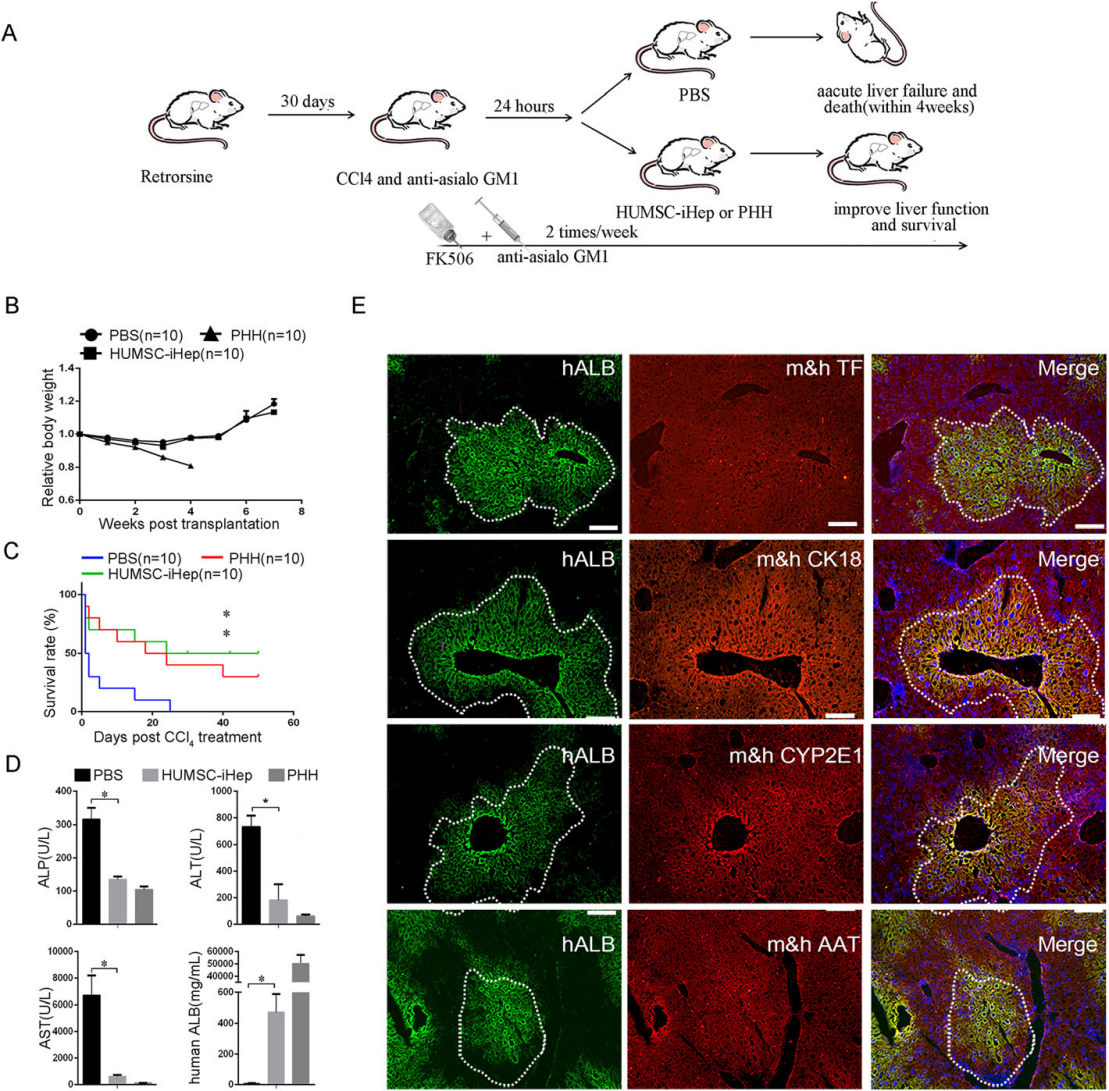
The therapeutic effects of HUMSC-iHeps on acute liver failure trigged by CCl4-retrorsine. **A** Schematic diagram of HUMSC-iHeps transplantation into the livers of CCl_4_/retrorsine-treated nude mice. Female BALB/c nude mice (approximately 7 weeks old) were pretreated with 70 mg/kg retrorsine at 30th and 14th days before cell transplantation. **B** Body weight of CCl_4_/retrorsine-treated nude mice that received no transplanted cells, PHHs, and HUMSC-iHeps. **C** Kaplan-Meier survival curves of CCl_4_/retrorsine-treated nude mice that received just PBS, HUMSC-iHeps, and PHHs. *P < 0.05, log rank test. **D** Serum levels of ALP, ALT, and AST in moribund control mice (*n* = 3), surviving HUMSC-iHeps-transplanted mice (*n* = 3), and surviving PHHs-transplanted mice (*n* = 3). Human ALB levels were determined by ELISA in the sera of surviving HUMSC-iHeps-transplanted and PHHs-transplanted mice. **E** Co-expression of human ALB and human-mouse chimeric antibody AAT, TF, CK18, and CYP2E1 in retrorsine-treated livers from mice transplanted HUMSC-iHeps measured by immunostaining

Expansion of HUMSC-iHeps in large numbers allowed the measurement of hepatic functions in vivo. We transplanted HUMSC-iHeps into Balb/c nude mice with acute liver failure trigged by CCl_4_-retrorsine (Fig. 5A).

The HUMSC-iHeps transplanted mice lost their body weights during the first 3 weeks but later regained body weight (Fig. 5B), indicating that transplanted HUMSC-iHeps restored functions of damaged livers. Besides, mice that underwent HUMSC-iHeps transplantation gained body weight nearly the same time as mice transplanted with PHHs, suggesting that powerful proliferation capability of HUMSC-iHeps to support liver function after transplantation (Fig. 5B).

All Balb/c nude mice without cell transplantation lost body weights and died within 4 weeks after CCl_4_ injection (Fig. 5B,C), whereas transplantation with PHHs and HUMSC-iHeps extended the life of Balb/c nude mice (Fig. 5C, 3 of 10 mice survived). Notably, 4 of the 10 mice transplanted with HUMSC-iHeps survived more than 9 weeks (Fig. 5C), showing similar survival curve as PHHs-transplanted mice.

Serum levels of ALP, ALT, and AST were significantly reduced in the surviving recipients from both PHHs-transplanted and HUMSC-iHeps-transplanted mice (Fig. 5D), further confirming the improved liver functions in these mice. Human ALB was also detected in the sera of mice transplanted with HUMSC-iHeps (Fig. 5D).

Immunohistochemical staining of human ALB showed that HUMSC-iHeps repopulated the liver parenchyma in the surviving mice (Fig. 5E). Repopulated HUMSC-iHeps were positively stained by m&h TF, m&h CK18, m&h CYP2E1, and m&h AAT antibody, signifying that engrafted HUMSC-iHeps exhibited a higher level of maturity (Fig. 5E).

**Fig. 6:**
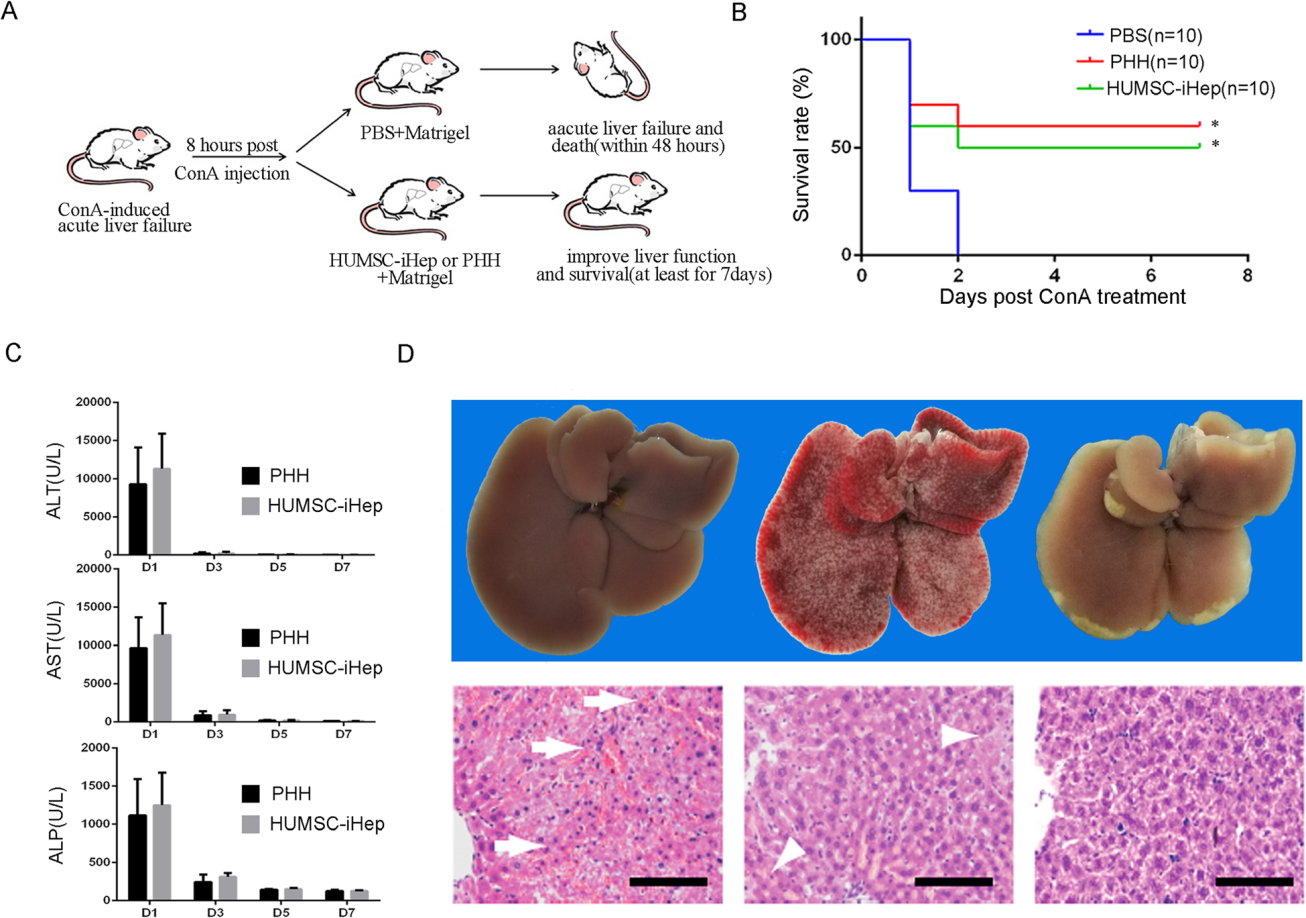
Transplantation of HUMSC-iHeps improve acute liver failure induced by concanavalin A. **A** Schematic diagram of encapsulated HUMSC-iHeps transplantation relieved mice with CoA-induced acute liver failure. **B** The Kaplan-Meier survival curves of CoA-induced acute liver failure mice that injected PBS, encapsulated HUMSC-iHeps, and PHHs. *P < 0.05, log rank test. **C** Serum levels of ALT, ALP, and AST in moribund control mice (*n* = 3), surviving HUMSC-iHeps-transplanted mice (n=3), and surviving PHHs-transplanted mice (*n* = 3). **D** Livers (up), macroscopic images of freshly isolated livers and liver sections (down), hematoxylin and eosin staining from Con-A-treated mice before (day 0), and after HUMSC-iHeps transplantation (days 4 and 7). Note Con-A-induced hepatitis and hemorrhage in the liver at day 0 (up) and arrows in (down), residual liver damage at day 4 (up) and arrowheads in (down), and the completely recovered liver at day 7

We next investigated whether HUMSC-iHeps could support liver in recovering from fulminant hepatitis in mice model. We injected concanavalin A (Con A) into wild-type mice to trigger fulminate hepatitis [18]. Eight hours after Con A treatment, we mixed HUMSC-iHeps, PHHs, and PBS solutions with Matrigel and intraperitoneally injected them into acute liver failure mice triggered by Con A. We used Matrigel for the reason that it is liquid at 4°C, but become a solid gel and could then encapsulate the cells after injected into mice with temperature of 37°C, protecting the engrafted cells from immunological rejection without obstructing the exchange of molecules (Fig. 6A). In groups transplanted with injected PBS, all mice died within 48 h after Con A treatment (Fig. 6B). PHHs treatment significantly raised survival rate and prolonged survival of mice with acute liver failure (Fig. 6B). Interestingly, upon treatment with encapsulated HUMSC-iHeps, 5 of 10 mice completely restored health from Con-A-induced acute liver failure (Fig. 6B) and showed normal serum ALP, ALT, and AST levels 3 days after Con A treatment (Fig. 6C). Histological analysis further confirmed that transplantation of HUMSC-iHeps profoundly improved liver damage caused by Con A (Fig. 6D). Together, these results revealed the hepatic functions of HUMSC-iHeps in vivo and provide evidence for the therapeutic effect of HUMSC-iHeps in treatment of liver injuries, for example, acute liver failure.

### In vivo safety assessment of HUMSC-iHeps

Considering that c-myc is a carcinogene, we further explore the safety of HLCs both in vitro and in vivo. We firstly analyzed the expression of a 13-gene signature, which significantly correlated with assays for telomerase enzymatic activity, including telomeric repeat amplification protocol (TRAP) assay and direct enzymatic assays, and thus could robustly inform telomerase enzymatic activity as reported by Nighat [19], from RNA-seq data of HUMSC-iHeps, HSF-iHeps, HUMSCs, and PHHs. It was obvious that 11 out of 13 genes showed much higher expression in HUMSC-iHeps and HSF-iHeps than that in HUMSCs and PHHs (Table S4), suggesting higher risk of tumorgenesis. However, the core subunits of telomerase TERT and TERC were lowly expressed or not expressed in both HUMSC-iHeps and HSF-iHeps (FPKM of TERT in HUMSC-iHeps and HSF-iHeps: 1.20, 1.00; FPKM of TERC in HUMSC-iHeps and HSF-iHeps: 0, 0.04).

To further assess the tumorigenic potential of HUMSC-iHeps, we subcutaneously inoculated HUMSC-iHeps or HepG2 into 10 nude mice. Two weeks later, subcutaneous tumors were observed in all mice inoculated HepG2. However, no tumor was detected in 10 mice inoculated HUMSC-iHeps in the long period of 3 months. This indicates that c-Myc induction does not raise the tumorigenic risk.

Moreover, karyotype analysis showed that HUMSC-iHeps maintained normal chromosome number and morphology at late passages (Figure S6A) and did not form tumors after transplantation in immunodeficient mice (Figure S6B), suggesting good security and potential in vivo application of our method.

### c-Myc acted at early stage of trans-differentiation via altered transcriptional regulatory network

To understand contribution of c-Myc to the reprogramming process, we need to figure out the role c-Myc act at different stages of the reprogramming. We reprogrammed HUMSCs with 3TFs and a doxycycline-inducible c-Myc (Figure S7A). Based on previous researches [20], we divided the whole induction process into three phases, with 6 days per phase. HUMSCs were exposed in Dox alternatively in 6 different combinations of reprogramming stages as showed in schematic diagram (Figure S7B). TTR-mCherry lentivirus was used to measure the trans-differentiation efficiency. Of the total 6 combinations, we found the highest proportion of red fluorescent cells in group I. We further discovered a pattern that the longer and the earlier c-Myc expressed, the higher the red fluorescent cell ratio was. This result revealed that c-Myc promoted reprogramming mainly at the early stage (0–6 days) (Figure S7C), which was consistent with result from the iPSCs induction [20].

To uncover the molecular mechanism underlying enhanced efficiency of c-Myc, we integrated RNA-seq with ChIP-seq to determine target genes of c-Myc in HUMSCs inducted by 3TFs+c-Myc and compared differential transcriptome profile of the two induction groups (3TFs vs 3TFs+c-Myc) using inducted HUMSCs 6 days post induction. This time point was chosen to define early transcriptional profiles that c-Myc functioned predominantly.

**Fig. 7:**
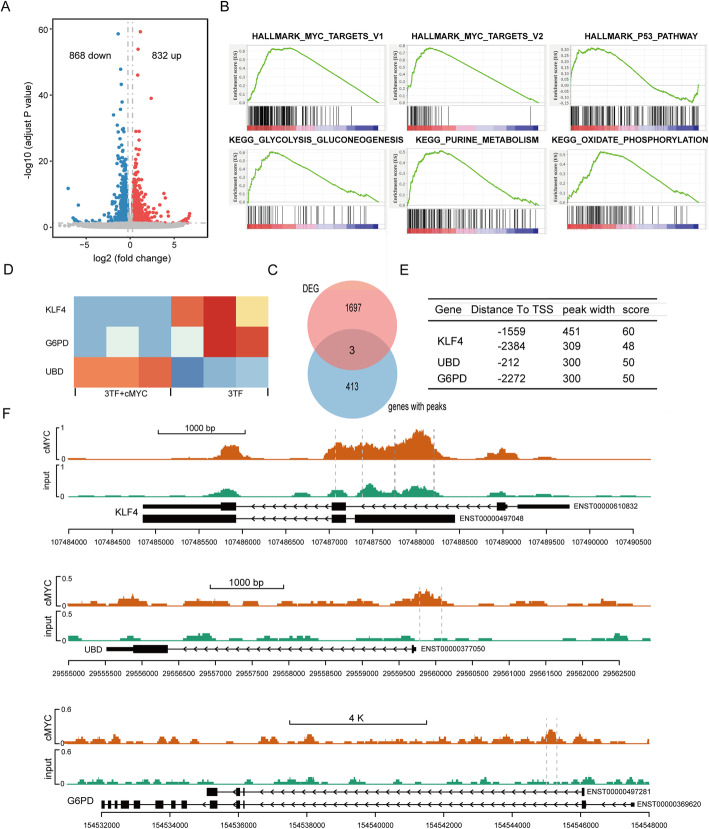
Regulatory network altered by c-Myc at early stage of induction revealed by RNA-seq and ChIP-seq. **A** Volcano plot indicating differentially expressed genes in the c-Myc+3TFs group relative to 3TFs-induced HUMSCs after 6 days of induction. **B** Enriched gene sets and pathways in c-Myc+3TFs induced HUMSCs revealed by GSEA analysis. **C** Three genes with c-Myc binding sites in their promoters expressed differentially in the two groups. **D** Heatmap of the three genes’ expression in six samples. **E** c-Myc binding site information of the three genes detected from ChIP-seq. **F** Genome browser plot showing the c-Myc binding sites within promoter of the three genes, KLF4, UBD, and G6PD

The experimental strategy is shown in Figure S8A. The two HUMSC groups with and without exogenous c-Myc stimulation were separated by unsupervised hierarchical clustering, suggesting their distinctive gene expression profiles (Figure S8B). Differential expression genes were identified if the following three conditions were satisfied: (i) mean expression FPKM value ≥0.5 in each group, (ii) absolute value of fold changes > 1.2, and (iii) adjusted *P* value <0.05. A total of 832 upregulated and 868 downregulated genes were obtained (Fig. 7A, Table S5). On total, 66 differentially expressed genes including SLC29A2, DUSP2, and SLC19A1 were c-Myc target genes as reported by previous research [21], highlighting the active regulatory role of c-Myc. Gene Set Enrichment Analysis (GSEA) unraveled that c-Myc caused significant promotion of MYC targets, E2F targets, and G2M checkpoint, which may contribute to the promotion effects of c-Myc on cell cycle and proliferation (Fig. 7B, Figure S8C). In addition, c-Myc consistently activated p53 pathway (Fig. 7B). Moreover, metabolic related pathways such as glycolysis and gluconeogenesis, oxidative phosphorylation, and purine metabolism, were significantly activated by c-Myc (Fig. 7B). The transcriptome divergence caused by c-Myc is consistent with the notion that c-Myc affects global gene regulatory networks with specific influence on metabolism, cell proliferation, and apoptosis [22]. Intriguingly, genes involved in epithelial mesenchymal transition (EMT) and cell adhesion were repressed by c-Myc (Figure S8D). This result is in line with the findings that a synchronous EMT occurs during the formation of definitive endoderm, while further differentiation of definitive endoderm to HLCs is accompanied by a mesenchymal to epithelial transition (MET) [23].

We performed ChIP-seq on HUMSCs infected by 3TFs+c-Myc (6 days post infection) in order to further determine direct target genes of c-Myc. We used DNA input as control for ChIP-seq. Binding sites of c-Myc were distributed mainly along intergenic region, gene body, and promoter (Figure S8E). We discovered 416 genes harbored at least one c-Myc binding site within their promoters (3kb surrounding TSS). To unravel the genes that c-Myc directly target to trigger differential transcriptome profiles, we analyzed genes that bounded by c-Myc and differentially expressed at the same time, and 3 genes (KLF4, UBD, G6PD) were identified (Fig. 7C–F). Among these four genes, KLF4 presented the highest score (Fig. 6E, F) and harbored two c-Myc binding sites within its promoter, highlighting its critical role in c-Myc regulatory network. It is known that KLF4 is a component of p53 pathway, suggesting c-Myc may promote reprogramming efficiency via transcriptional regulation of apoptosis. Of course, more evidences will be needed to draw a conclusion. We will do knockdown experiment to demonstrate the direct regulatory relationship between c-Myc and KLF4 and unravel the corresponding phenotypic alteration in further research.

## Discussion

Large quantities of functional liver cells are needed for liver regenerative medicine [2]. Although much progress has been made to expand primary human hepatocytes in vitro [3, 5], it still cannot meet demand of clinical hepatocyte transplantation because of the immune rejection and donor shortage [2]. HLCs derived from direct lineage reprogramming are one of the most promising solutions. Currently, source cells used for direct reprogramming of HLCs include fibroblasts and endothelial cells [13, 24], which are cells with poor expansion capabilities. HUMSCs derived from neonatal umbilical cords, have the advantages of easy acquisition, low immunogenicity, and strong expansion capacity in vitro [16]. However, no direct reprogramming protocol of converting HUMSC to HLCs has been reported.

In this study, we successfully generated functional HLCs from HUMSCs by forced expression of the transcription factors FOXA3, HNF1A, and HNF4A. What is more, we found c-Myc greatly promoted the trans-differentiation efficiency. HUMSC-iHeps exhibited an expandable bi-phenotypic status, which expressed markers of both mature hepatocytes and liver progenitors. Notably, these cells could be serially expanded about 10^6^-fold in 30 days. HUMSC-iHeps retained some of the functional characteristics of mature hepatocytes, including plasma protein secretion, glycogen storage, and lipid accumulation. HUMSC-iHeps expressed CYP450 enzymes, which were crucial for drug metabolism in mature hepatocytes, although their expression levels were not as high as those of PHHs. Kong et al. recently reported that the combined transplantation of human primary hepatocytes and mesenchymal stem cells is considered to be more effective for the treatment of mouse acute liver failure than single-cell transplantation [25]. Considering HUMSC-iHeps has expression profiles of both progenitor and mature hepatocytes, we believe that HUMSC-iHeps may have better clinical therapeutic potential.

Previous protocols showed about 10,000-fold expansion of human hepatocytes in vitro at P8 without any genetic manipulation [3], whereas HUMSC-iHeps in this study can only expand about 10^6^-fold in 30 days in vitro. Hepatocyte transplantation is considered as a promising alternative treatment to liver transplantation, and the number of cells transplanted for one patient is around 10^9^ [26]. As a proof of principle, our study could provide sufficient hepatocyte-like cells in short time, which may be further developed as a cellular source for the treatment of liver diseases.

HUMSC-iHeps possessed strong proliferation capacity with the cell doubling time of 2.5 days for both P4 and P10 in vitro. In addition, repopulation of HUMSC-iHeps was able to rescue 40–50% mice with CCl_4_-retrorsine-induced and CoA-induced acute liver failure, demonstrating liver damage restore function of these cells.

Although c-Myc is frequently used in trans-differentiation protocols to generate HLCs [14, 27, 28], the molecular mechanism is still elusive. We found c-Myc enhanced 3TFs-mediated reprogramming of HUMSCs mainly at the early stage (0–6 days), which was consistent with results reported in iPSCs reprogramming [20]. To uncover the regulatory network that responded to c-Myc in HUMSCs, gene expression data from HUMSCs inducted with 3TFs and 3TFs+c-Myc were obtained by RNA-seq. Consistently, GSEA result revealed significant enrichment on gene sets of HALLMARK_MYC_TARGETS_V1 and HALLMARK_MYC_TARGETS_V2, which consist of a collection of well-characterized genes whose transcription is directly regulated by MYC. Notably, we found EMT were repressed in 3TFs+c-Myc group, which was supported by the findings that differentiation of definitive endoderm to HLCs is accompanied by a mesenchymal to epithelial transition (MET) [23]. We further used ChIP-seq to identify direct target genes of c-Myc, and we obtained 416 c-Myc binding genes. What is more, 3 genes (KLF4, UBD, G6PD) were found also differentially regulated by c-Myc, among which KLF4 presented the highest score and harbored two c-Myc binding sites within its promoter, highlighting the critical role of KLF4 in c-Myc-mediated HLCs induction. We will further demonstrate the molecular relationship of c-Myc and KLF4, UBD, and G6PD by knockdown experiment of c-Myc.

## Conclusions

In summary, this study unraveled that c-Myc enhanced the reprogramming and trans-differentiation of 3TFs-induced HUMSCs and fibroblasts into HLCs. HUMSC-iHeps retained some of the functional characteristics of mature hepatocytes, including plasma protein secretion, glycogen storage, and lipid accumulation. However, the maturation of HUMSC-iHeps was incomplete, which required culture system to be further developed to promote their maturation. HUMSC-iHeps could rescue mice with acute liver injury, and in vivo safety assessment of HUMSC-iHeps revealed no tumorigenic risk, suggesting restore function and good security of HUMSC-iHeps. In conclusion, our protocol for HLCs provides insights into c-Myc promotion and a source of cells for therapeutic applications.

## Supplementary Information

**Additional file 1: Table S1.** Primer sequences of genes used in this study. **Table S2.** Nuber of epithelial-like cell clones. **Table S3.** primer sequence of si-cMYC. **Table S4.** Expression of 13-gene signature for telomerase enzymatic activity (FPKM). **Table S5.** Differentially expressed genes between 3TFs and 3TFs+cMYC.

**Additional file 2: Figure S1.** Characterization of HUMSCs. (A) Morphology of Primary HUMSCs (P0), HUMSCs in passage 5 (P5) and HUMSCs in passage 10 (P10). The cells showed homogeneous fibroblastic morphology. (B) Expression of cell surface markers on HUMSCs. HUMSCs were positive for mesenchymal stem markers (CD166, CD29,CD90, CD105 and CD44) and negative for hematopoietic marker (CD45, CD34 and HLA-DR) or endothelial marker (CD31). (D) Multiple differentiation potential of HUMSCs. HUMSCs could differentiate into adipocytes, osteocytes and chondrocytes. Scale bar: 50 μm (A) 100 μm (D). **Figure S2.** HSFs-iHeps harbored hepatocyte characteristics.(A) cell morphology of HSF, HSF-iheps and PHHs. (B) Hepatic gene expression in HSF-iHeps detected by western blot.(C) Expression of hepatic genes in HSF-iHeps measured by qPCR. (D) Co-expression of ALB and HNF4A, TF, AAT, MRP2, CK18 and ASS1 in HSF-iHeps revealed by immunofluorescence staining. **Figure S3.** HSF-iHeps possessed hallmark functions of mature hepatocytes. (A) Basic liver function analysis of HSF-iHep, including oil red O staining (i) PAS staining (ii) uptake of ICG (iii) LDL uptake. (B) ELISA was used to detect the secretion of ALB (left) and (C) AAT (right) during hepatic induction of HSF-iHeps. (D) The mRNA levels of CYP genes were determined by qPCR in PHHs and HSF-iHeps cultured for 2 days before inducer treatment. Data are normalized to PHHs. (E) The mRNA levels of the induced CYP enzymes were measured by qPCR. Data are represented as the mean ± SD. (F) Expression of drug transporter genes in HSF-iHeps determined by qPCR. Data are normalized to PHHs. **Figure S4.** Transcriptome pattern of HUMSC-iHep cells. (A) Whole-genome expression analysis shows the gene expression of HUMSC, PHH, HUMSC-iHep, HSF-iHep, Hepatoblast and Fetal liver cells. HUMSC-iHep and HSF-iHep are grouped with hepatoblast, but closer to Fetal liver more than PHH, as shown in the cluster tree.Color representation represents the level of expression.(B) Heat maps of the expression of fibroblast genes, hepatic transcription factors, functional hepatocyte genes involved in glucose metabolism, lipid cholesterol metabolism, fatty acid metabolism, coagulation and drug metabolism in various cells. (C) qPCR results showed that the functional genes of the mature bile duct, such as cystic fibrosis transmembrane conduction regulator (CFTR), secretin receptor (SCTR), somatostatin receptor 2 (SSTR2), and aquaprin-1 (AQP1), are not expressed in HUMSC-iHeps, suggesting that the cells are heterogeneous but do not differentiate into bile duct epithelial cells. Data are normalized to PHHs. **Figure S5.** Gene Set Enrichment Analysis (GSEA) of HUMSC-iHeps and PHHs. GSEA analysis revealed remarkably enriched hepatic gene expression in both PHHs and HUMSC-iHeps. Gene sets were compiled from BIOCARTA, the Kyoto Encyclopedia of Genes and Genomes (KEGG). A peak shift to the left side indicates the enrichment of the indicated set of hepatic genes in PHHs or HUMSC-iHeps. **Figure S6.** In vivo safety assessment. (A) HUMSC-iHep (2×10^6^) and HepG2 (2×10^6^) cells were subcutaneously transplanted into the flank areas of nude mice. HUMSC-iHeps did not form tumors 3 months after transplantation. But HepG2 cells form tumors 2 weeks after transplantation. (B) The karyotypes of HUMSC-iHep cells at passage 10 were analyzed by chromosome analysis during mitosis. **Figure S7.** c-Myc promoted reprogramming mainly at the early stage (0-6 days). (A) c-Myc levels in HUMSCs cells before and after induction with doxycycline (Dox) as measured by western blot. (B) Six combinations of doxycycline-induced c-Myc expression during the reprogramming of HUMSCs into HLCs. The whole induction 18 days were divided into 3 phases. Red bars represent the DOX addition and gray bars represent Dox withdrawn. (C) The proportion of red fluorescent cells in six groups was detected by flow cytometry after induction for 18 days. **Figure S8.** Regulatory network altered by c-Myc. (A) Schematic diagram depicts the experiment strategy. (B) Transcriptome correlation analysis of HUMSCs inducted by 3TFs(KT1-3) and 3TFs+c-Myc(CKT1-3). (C) Expression of genes from two gene sets: E2F targets and G2M checkpoint in two groups of HUMSCs inducted by 3TFs(KT1-3) and 3TFs+c-Myc(CKT1-3). (D) GSEA enrichment plot of two gene sets. (E) Genomic distribution of c-Myc binding sites.

## Data Availability

The publicly available data sets (Accession Numbers: GEO: GSE101133, GSE128102) were also used in this study. The datasets used and/or analyzed during the current study are available on China National GeneBank (Accession Numbers: CNP0001776).

## Abbreviations

HUMSCs: Human umbilical cord mesenchymal stem cells
HLCs: Hepatocyte-like cells
HUMSC-iHeps: Hepatocyte-like cells derived from human umbilical cord mesenchymal stem cells
iPSCs: Induced pluripotent stem cells
HSFs: Human skin fibroblasts
HSC-iHeps: Hepatocyte-like cells derived from human skin fibroblasts
PHHs: Primer human hepatocytes
3TFs, FOXA3, HNF1A, HNF4A: c-Myc, MYC proto-oncogene
GSEA: Gene Set Enrichment Analysis
KEGG: Kyoto Encyclopedia of Genes and Genomes
ChIP-seq: Chromatin immunoprecipitation sequencing
RNA-seq: RNA sequencing
ALB: Albumin
AAT: Alpha-1-antitrypsin
CYP: Cytochrome P450
qPCR: Quantitative polymerase chain reaction
FOXA3: Forkhead box protein A3
HNF1A: Hepatocyte nuclear factor 1a
HNF4A: Hepatocyte nuclear factor 4a
MET: Mesenchymal-epithelial transition
EMT: Epithelial-mesenchymal transition
PBS: Phosphate-buffered saline
IP: Immunoprecipitation
ChIP: Chromatin immunoprecipitation
DMEM: Dulbecco’s modified Eagle’s medium
ICG: Indocyanine green
LDL: Low-density lipoproteins
PAS: Periodic acid-Schiff stain
DPDPE: [D-Pen2,5]-Enkephalinhydrate
D8-TCA: d8-Taurocholate
CLF: Cholyl-lysyl-fluorescein
Dox: Doxycycline
BSA: Bovine serum albumin
FBS: Fetal bovine serum
HBSS: Hank’s balanced salt solution
ELISA: Enzyme-linked immunosorbent assay
