# Supplementary material for: High-efficiency c-Myc-mediated induction of functional hepatoblasts from the human umbilical cord mesenchymal stem cells
Source: Stem Cell Res Ther. 2021 Jul 2;12:375. doi: 10.1186/s13287-021-02419-1 (PMC8254319; doi:10.1186/s13287-021-02419-1)
Supplement: Supplementary file 2 — Additional file 2: Figure S1. Characterization of HUMSCs. (A) Morphology of Primary HUMSCs (P0), HUMSCs in passage 5 (P5) and HUMSCs in passage 10 (P10). The cells showed homogeneous fibroblastic morphology. (B) Expression of cell surface markers on HUMSCs. HUMSCs were positive for mesenchymal stem markers (CD166, CD29,CD90, CD105 and CD44) and negative for hematopoietic marker (CD45, CD34 and HLA-DR) or endothelial marker (CD31). (D) Multiple differentiation potential of HUMSCs. HUMSCs could differentiate into adipocytes, osteocytes and chondrocytes. Scale bar: 50 μm (A) 100 μm (D). Figure S2. HSFs-iHeps harbored hepatocyte characteristics.(A) cell morphology of HSF, HSF-iheps and PHHs. (B) Hepatic gene expression in HSF-iHeps detected by western blot.(C) Expression of hepatic genes in HSF-iHeps measured by qPCR. (D) Co-expression of ALB and HNF4A, TF, AAT, MRP2, CK18 and ASS1 in HSF-iHeps revealed by immunofluorescence staining. Figure S3. HSF-iHeps possessed hallmark functions of mature hepatocytes. (A) Basic liver function analysis of HSF-iHep, including oil red O staining (i) PAS staining (ii) uptake of ICG (iii) LDL uptake. (B) ELISA was used to detect the secretion of ALB (left) and (C) AAT (right) during hepatic induction of HSF-iHeps. (D) The mRNA levels of CYP genes were determined by qPCR in PHHs and HSF-iHeps cultured for 2 days before inducer treatment. Data are normalized to PHHs. (E) The mRNA levels of the induced CYP enzymes were measured by qPCR. Data are represented as the mean ± SD. (F) Expression of drug transporter genes in HSF-iHeps determined by qPCR. Data are normalized to PHHs. Figure S4. Transcriptome pattern of HUMSC-iHep cells. (A) Whole-genome expression analysis shows the gene expression of HUMSC, PHH, HUMSC-iHep, HSF-iHep, Hepatoblast and Fetal liver cells. HUMSC-iHep and HSF-iHep are grouped with hepatoblast, but closer to Fetal liver more than PHH, as shown in the cluster tree.Color representation represents the level of expression.( [file 13287_2021_2419_MOESM2_ESM.docx]

**Supporting Information**

**Supplementary Methods**

**Flow cytometry analysis**

HUMSCs were characterized using cell surface markers by fluorescence-activated cell sorting (FACS) analysis. 5×10^5^ HUMSCs (in 100 μL PBS) were incubated with different fluorescently labeled monoclonal antibodies (anti-rat CD45- PECy5, anti-rat CD31- PE, anti-rat CD29- FITC, anti-rat CD44H-FITC and anti-rat CD 90- FITC, Biolegend) and incubated in the dark at 2∼8℃ for 30 min. After washing with PBS twice, cells were resuspended in 300 μl PBS and analyzed by the Calibur flow cytometer (BD).

For intracellular staining of albumin and a-1-antitrypsin, 5×10^5^ iHeps were harvested and fixed with 4% PFA for 30 min, and then permeabilized in staining buffer (BD) for 10 min. Cells were then incubated with primary antibody (sheep anti-albumin, Bethyl; rabbit anti-alpha-antitrypsin, American Research Products) for 30 min in staining buffer, followed with secondary antibody (dylight 488 conjugated donkey anti-rabbit IgG, Bethyl; dylight 594 conjugated donkey anti-sheep IgG, Bethyl) incubation for 30 min. Cells were analyzed by the Calibur flow cytometer (BD).

**Adipogenic Differentiation**

Passage 3 HUMSCs were counted and seeded at a density of 10^5^ per well in a 6-well plate. When ∼100% confluent, adipogenesis differentiation media, which consists of high glucose DMEM (Gibco) supplemented with 10% FBS, 500μM isobutylmethylxanthine (IBMX), 1 μM dexamethasone, 200 μ M Indomethacin, 5μg/ml insulin (Sigma) was added to 4 wells; and complete culture Media was added to other 2 wells as negative controls. The medium was changed every 3 days for 2 weeks. The differentiation potential for adipogenesis and formation of intracellular lipid droplets were assessed by Oil-red-O staining.

**Osteogenic Differentiation**

Passage 3 HUMSCs were harvested by trypsin digestion as described above; the cells were counted and seeded at a density of 105 per well in a 6-well plate. When ∼100% confluent, osteogenesis differentiation media, which consists of high glucose DMEM (Gibco) supplemented with 10% FBS, 10 mM β-glycerol phosphate (Sigma), 50 μ M Ascorbic acid (Sigma), 0.1 μ M dexamethasone (Sigma), was added to 4 wells whereas complete culture Media was added to other 2 wells as negative controls. The medium was changed every 3 days for 3 weeks. The differentiation potential for osteogenesis was assessed by 40 mM Alizarin Red (pH 4.2) staining.

**Chondrogenic Differentiation**

Passage 3 MSCs were counted and seeded at a density of 106 per well in ultralow attachment 6-well plate. When the pellet cultures contain ∼ 2.5×105 HUMSCs, chondrogenic differentiation medium, which consists of high glucose DMEM (Gibco) supplemented with 10 ng/ml TGF-β3, 100nM dexamethasone, 200μM ascorbate-2- phosphate, 40 μ g/ml proline, 1 mM pyruvate, 1 mg/ml BSA and 50mg/ml ITS, was added to 4 wells and complete culture Media was added to other 2 well as negative controls. The medium was replaced every 3 days for 21 days. The differentiation potential for chondrogenesis was measured by Alcian blue staining.

**Induction period Medium Contents**

Reprograming medium is DMEM/F12 (Hyclone) supplemented with 0.544mg/L ZnCl_2_ (Sigma-Aldrich), 0.75mg/L ZnSO_4_·7H_2_O (Sigma-Aldrich), 0.2 mg/L CuSO_4_·5H_2_O (Sigma-Aldrich), 0.025mg/L MnSO_4_ (Sigma-Aldrich), 2g/L Bovine serum albumin (Sigma-Aldrich), 2g/L Galactose (Sigma-Aldrich), 0.1g/L Ornithine, 0.03g/L Proline, 0.61g/L Nicotinamide, 1X Insulin-transferrin-sodium selenite media supplement (Corning), 40 ng/ml TGFα (Peprotech), 40 ng/ml EGF (Peprotech), 10μM dexamethasone and 10% fetal bovine serum (Gibco), 1%NEAA, 1% Glutamax, 3µM CHIR99021, 2 µM A-83-01, 10 µM KU55933, 0.1 mM NaB, 0.5 µM RG-108, 2 µM tranylcpromine.

Hepatogenic medium is DMEM/F12 (Hyclone) supplemented with 0.544mg/L ZnCl_2_, (Sigma-Aldrich), 0.75mg/L ZnSO_4_·7H_2_O (Sigma-Aldrich), 0.2 mg/L CuSO_4_·5H_2_O (Sigma-Aldrich), 0.025mg/L MnSO_4_ (Sigma-Aldrich), 2g/L Bovine serum albumin (Sigma-Aldrich), 2g/L Galactose (Sigma-Aldrich), 0.1g/L Ornithine, 0.03g/L Proline, 0.61g/L Nicotinamide, 1X Insulin-transferrin-sodium selenite media supplement (Corning), 40 ng/ml TGFα (Peprotech), 40 ng/ml EGF (Peprotech), 10μM dexamethasone and 10% fetal bovine serum (Gibco), 1%NEAA, 1% Glutamax, 3µM CHIR99021, 2 µM A-83-01, 10 µM Dexamethasone, 0.61g/L Nicotinamide.

**PAS staining, DiI-LDL and ICG uptake assays, ALB ELISA, urea synthesis**

Induced cells were stained by Periodic-Acid-Schiff (PAS, Sigma) following the manufacturer’s instructions. For LDL uptake assay, cells were incubated with 10 mg/mL DiI-labeled LDL (Life Technologies) at 37°C for 1 hr and later washed with PBS three times. For indocyanine green (ICG, Sigma) uptake assay, medium of iHeps were changed with 1 mg/mL ICG and incubated at 37℃ for 1 hour, followed by washing with PBS three times.

To determine ALB secretion in iHeps, culture supernatants were collected daily and determined by the human albumin ELISA kit (Bethyl) according to the manufacturer’s instructions.

**Doxycycline-inducible c-Myc expression and shRNA-mediated gene silencing**

HUSMCs cells were stably transfected with a doxycycline-inducible two-vector system c-Myc (tet-on; Clontec Laboratories).Where indicated, c-Myc expression was induced by addition of doxycycline (30 hour; 1 mg ml21). Lentiviruses expressing an shRNA against c-Myc were generated using a pLKO vector (TRCconsortium) by co-transfection with the packaging plasmid psPAX.2, and the envelope plasmid pMD2.G into HEK293 cells. Cells were infected with lentiviral supernatants in the presence of 4 mg/mL polybrene (Sigma) for 24 hours. Infected cells were harvested 72~96 hours after infection for analysis.

**Supplementary Figures**


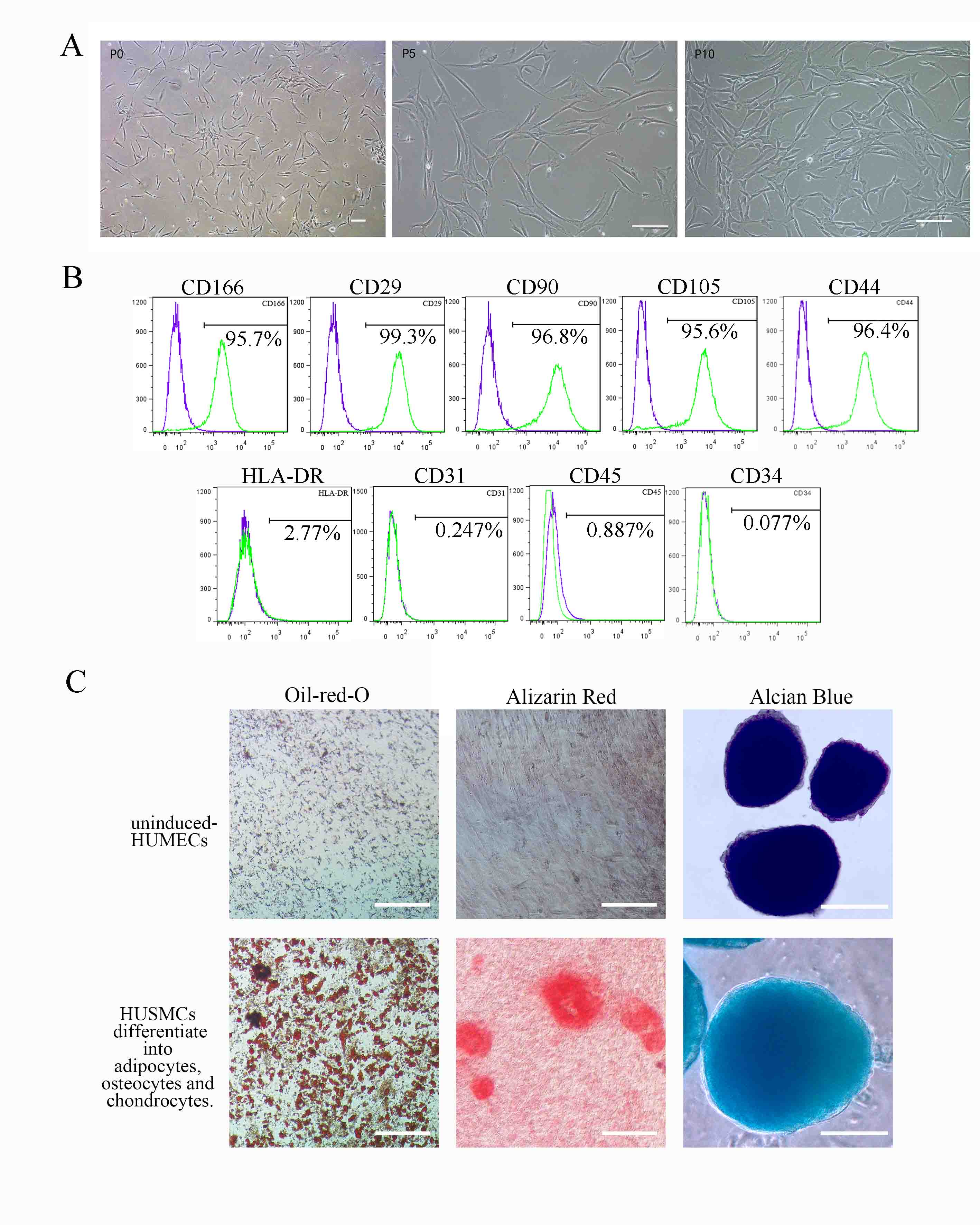


Figure. S1. Characterization of HUMSCs. (A) Morphology of Primary HUMSCs (P0), HUMSCs in passage 5 (P5) and HUMSCs in passage 10 (P10). The cells showed homogeneous fibroblastic morphology. (B) Expression of cell surface markers on HUMSCs. HUMSCs were positive for mesenchymal stem markers (CD166, CD29,CD90, CD105 and CD44) and negative for hematopoietic marker (CD45, CD34 and HLA-DR) or endothelial marker (CD31). (D) Multiple differentiation potential of HUMSCs. HUMSCs could differentiate into adipocytes, osteocytes and chondrocytes. Scale bar: 50 μm (A) 100 μm (D).


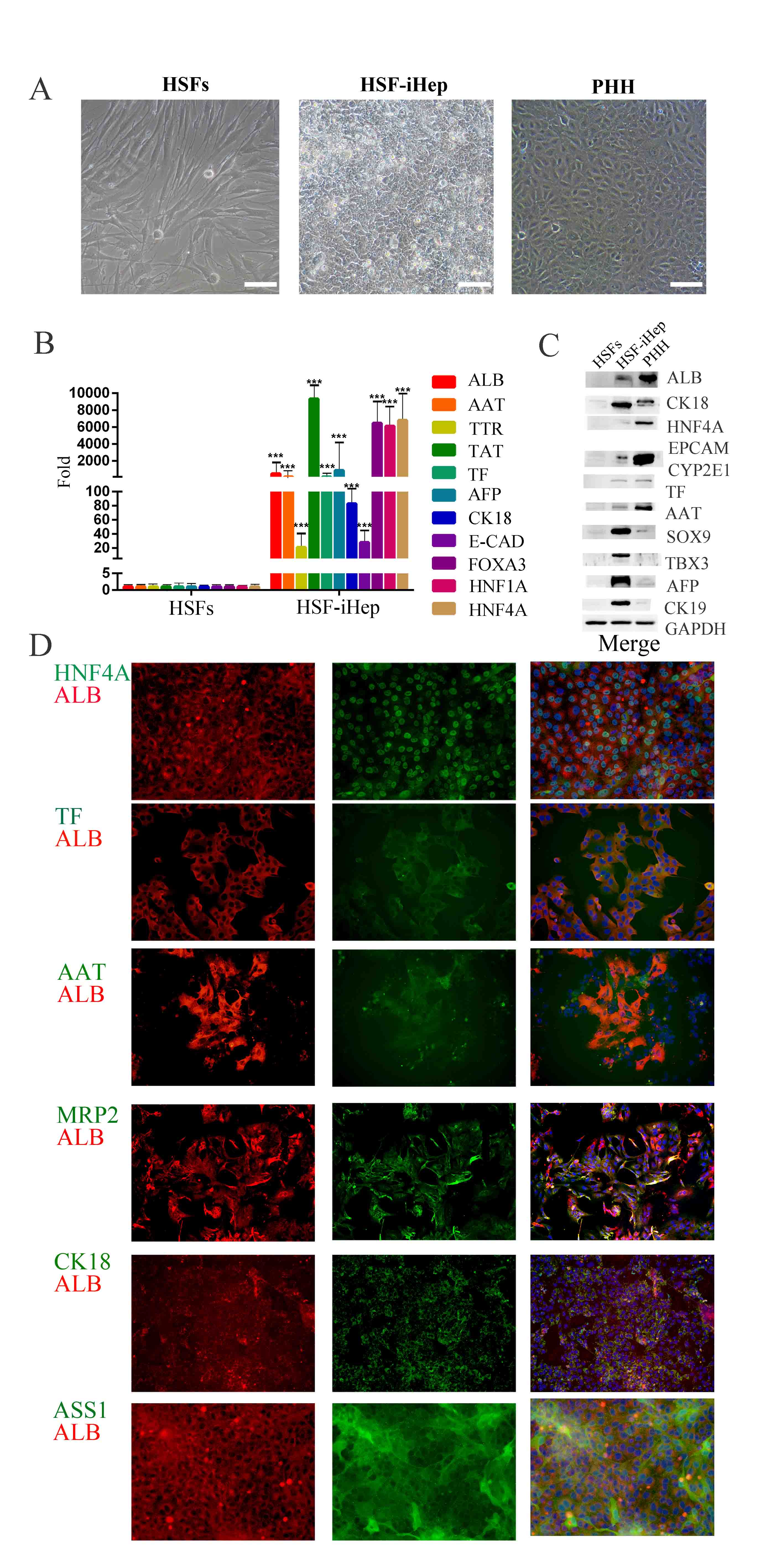


Figure S2. HSFs-iHeps harbored hepatocyte characteristics.(A) cell morphology of HSF, HSF-iheps and PHHs. (B) Hepatic gene expression in HSF-iHeps detected by western blot.(C) Expression of hepatic genes in HSF-iHeps measured by qPCR. (D) Co-expression of ALB and HNF4A, TF, AAT, MRP2, CK18 and ASS1 in HSF-iHeps revealed by immunofluorescence staining.


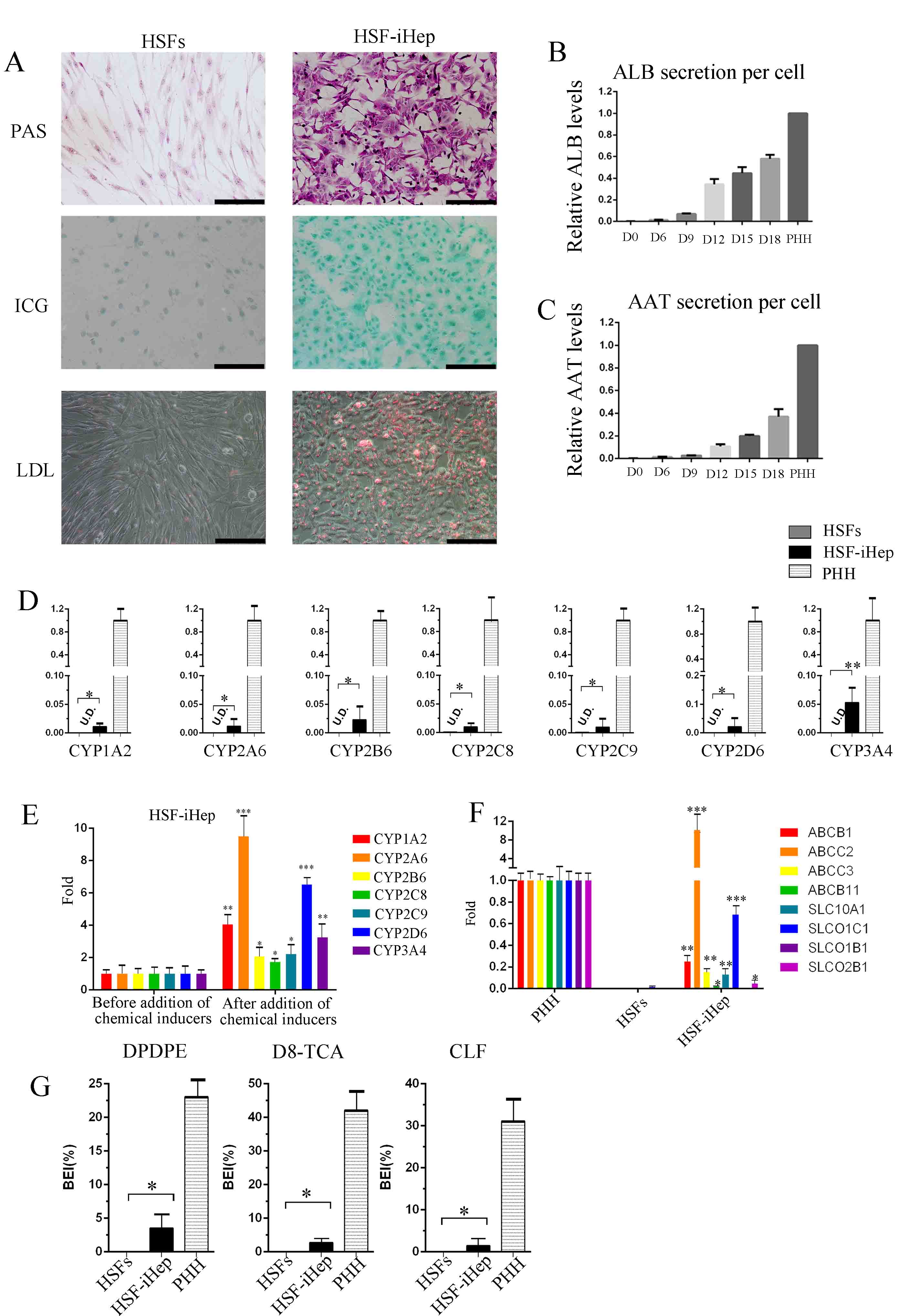


Figure S3. HSF-iHeps possessed hallmark functions of mature hepatocytes. (A) Basic liver function analysis of HSF-iHep, including oil red O staining (i) PAS staining (ii) uptake of ICG (iii) LDL uptake. (B) ELISA was used to detect the secretion of ALB (left) and (C) AAT (right) during hepatic induction of HSF-iHeps. (D) The mRNA levels of CYP genes were determined by qPCR in PHHs and HSF-iHeps cultured for 2 days before inducer treatment. Data are normalized to PHHs. (E) The mRNA levels of the induced CYP enzymes were measured by qPCR. Data are represented as the mean ± SD. (F) Expression of drug transporter genes in HSF-iHeps determined by qPCR. Data are normalized to PHHs.


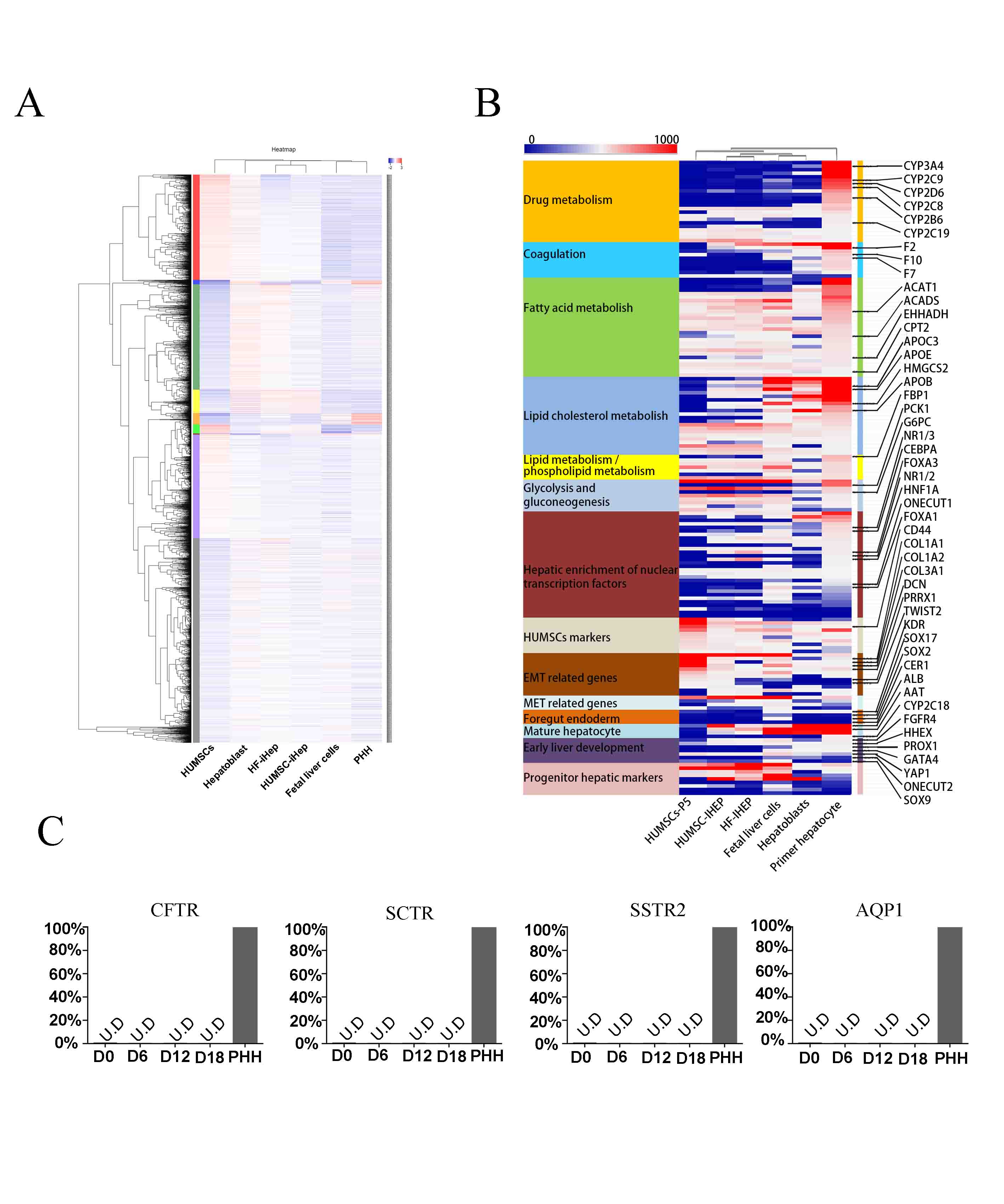


Figure S4. Transcriptome pattern of HUMSC-iHep cells. (A) Whole-genome expression analysis shows the gene expression of HUMSC, PHH, HUMSC-iHep, HSF-iHep, Hepatoblast and Fetal liver cells. HUMSC-iHep and HSF-iHep are grouped with hepatoblast, but closer to Fetal liver more than PHH, as shown in the cluster tree.Color representation represents the level of expression.(B) Heat maps of the expression of fibroblast genes, hepatic transcription factors, functional hepatocyte genes involved in glucose metabolism, lipid cholesterol metabolism, fatty acid metabolism, coagulation and drug metabolism in various cells. (C) qPCR results showed that the functional genes of the mature bile duct, such as cystic fibrosis transmembrane conduction regulator (CFTR), secretin receptor (SCTR), somatostatin receptor 2 (SSTR2), and aquaprin-1 (AQP1), are not expressed in HUMSC-iHeps, suggesting that the cells are heterogeneous but do not differentiate into bile duct epithelial cells. Data are normalized to PHHs.


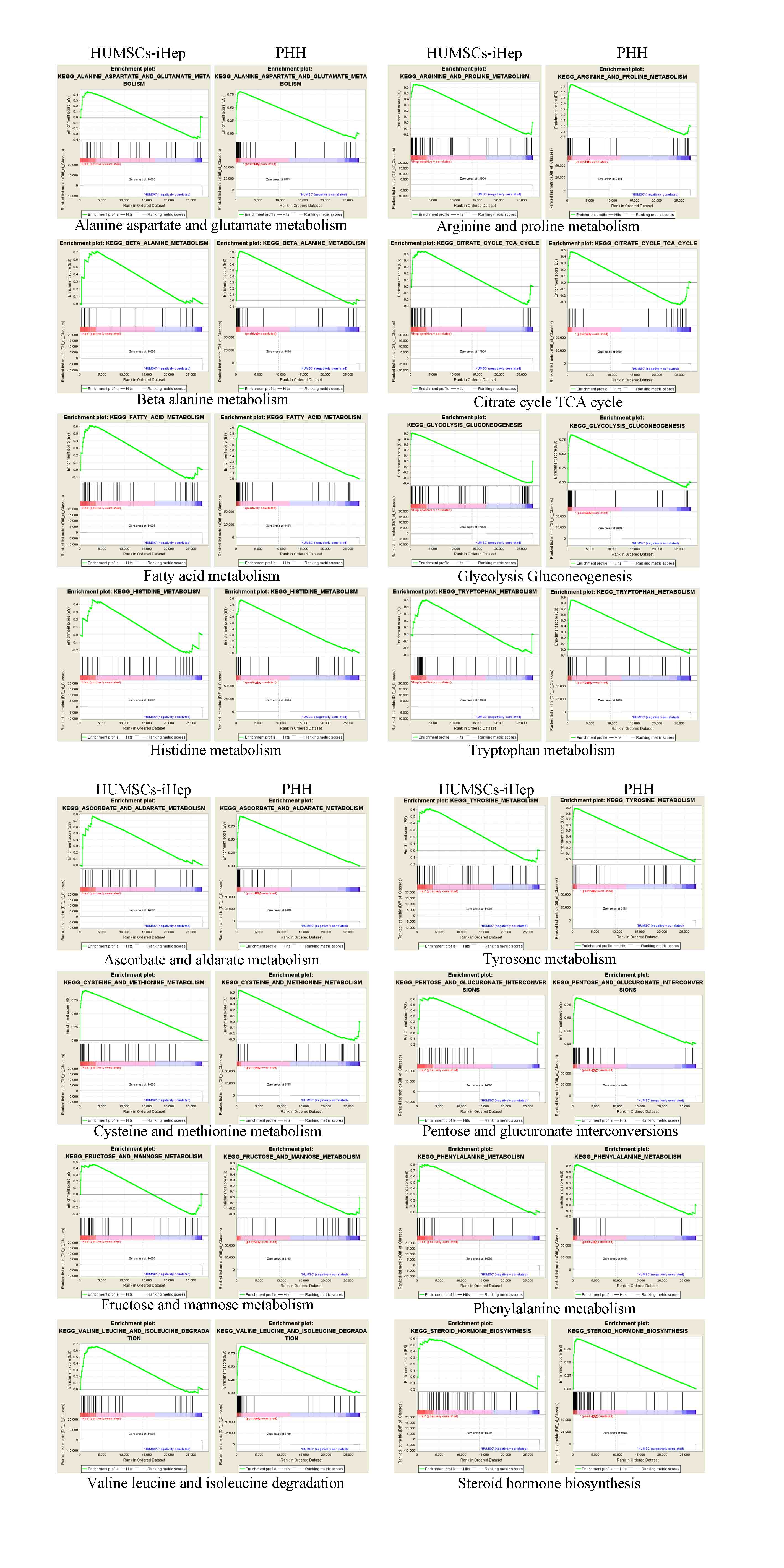


Figure S5. Gene Set Enrichment Analysis (GSEA) of HUMSC-iHeps and PHHs. GSEA analysis revealed remarkably enriched hepatic gene expression in both PHHs and HUMSC-iHeps. Gene sets were compiled from BIOCARTA, the Kyoto Encyclopedia of Genes and Genomes (KEGG). A peak shift to the left side indicates the enrichment of the indicated set of hepatic genes in PHHs or HUMSC-iHeps.


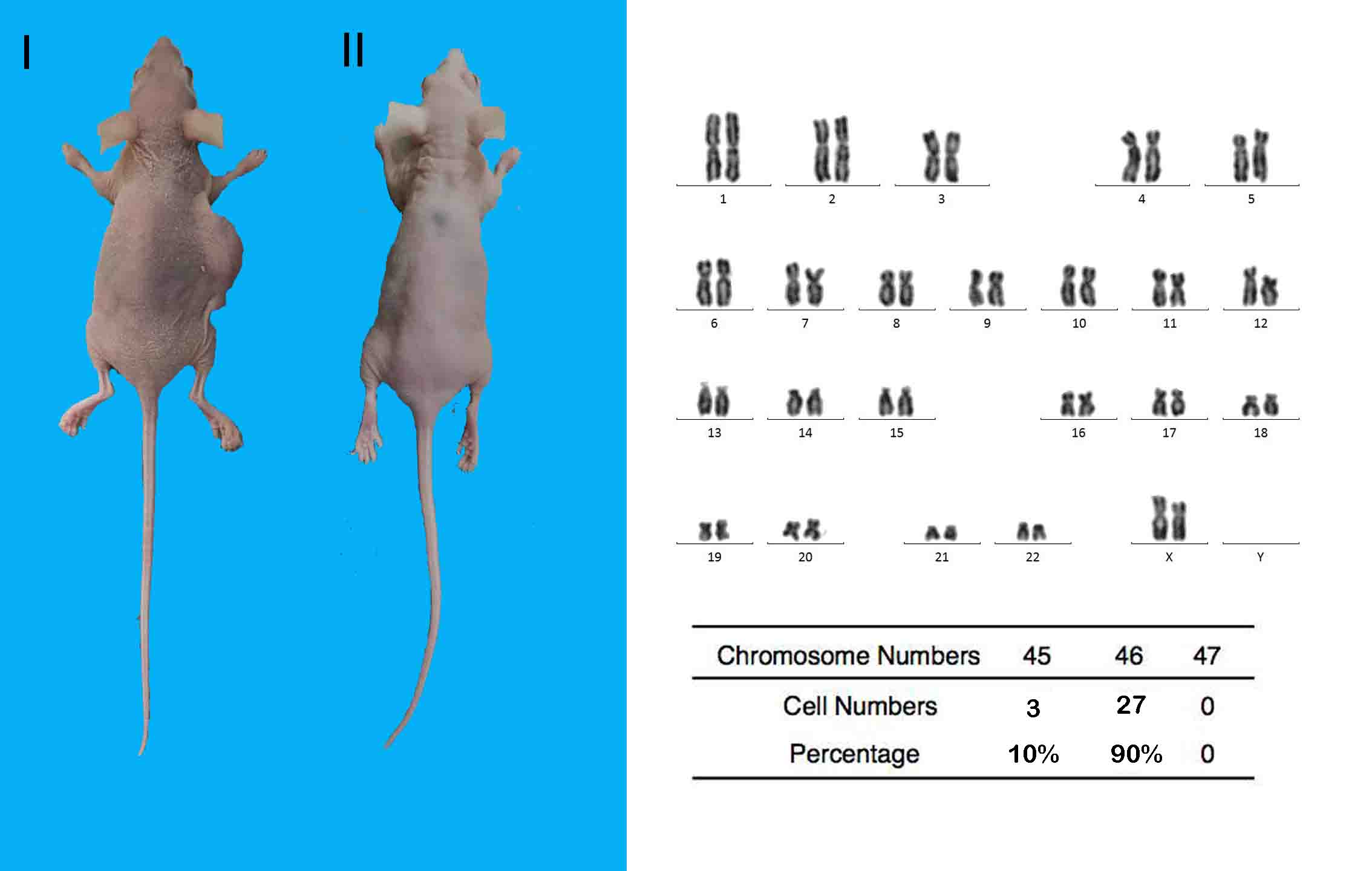


Figure S6. In vivo safety assessment. (A) HUMSC-iHep (2×10^6^) and HepG2 (2×10^6^) cells were subcutaneously transplanted into the flank areas of nude mice. HUMSC-iHeps did not form tumors 3 months after transplantation. But HepG2 cells form tumors 2 weeks after transplantation. (B) The karyotypes of HUMSC-iHep cells at passage 10 were analyzed by chromosome analysis during mitosis.


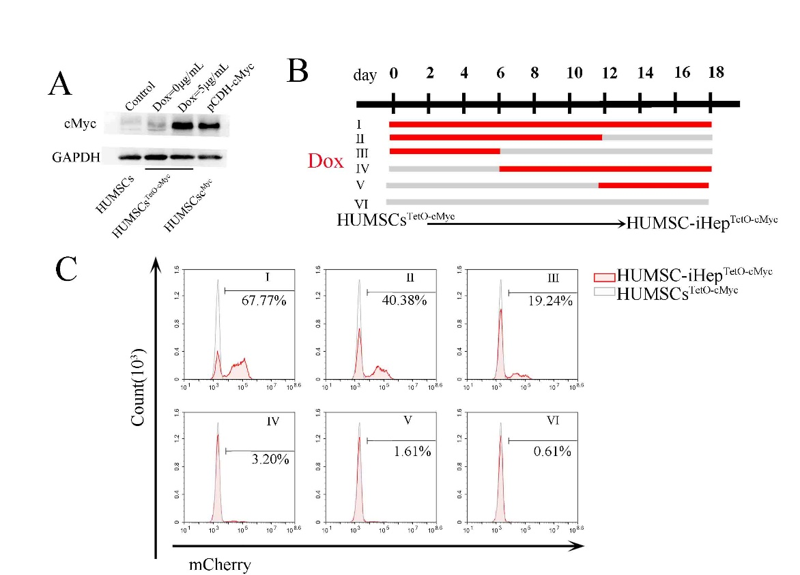


Figure S7. c-Myc promoted reprogramming mainly at the early stage (0-6 days). (A) c-Myc levels in HUMSCs cells before and after induction with doxycycline (Dox) as measured by western blot. (B) Six combinations of doxycycline-induced c-Myc expression during the reprogramming of HUMSCs into HLCs. The whole induction 18 days were divided into 3 phases. Red bars represent the DOX addition and gray bars represent Dox withdrawn. (C) The proportion of red fluorescent cells in six groups was detected by flow cytometry after induction for 18 days.


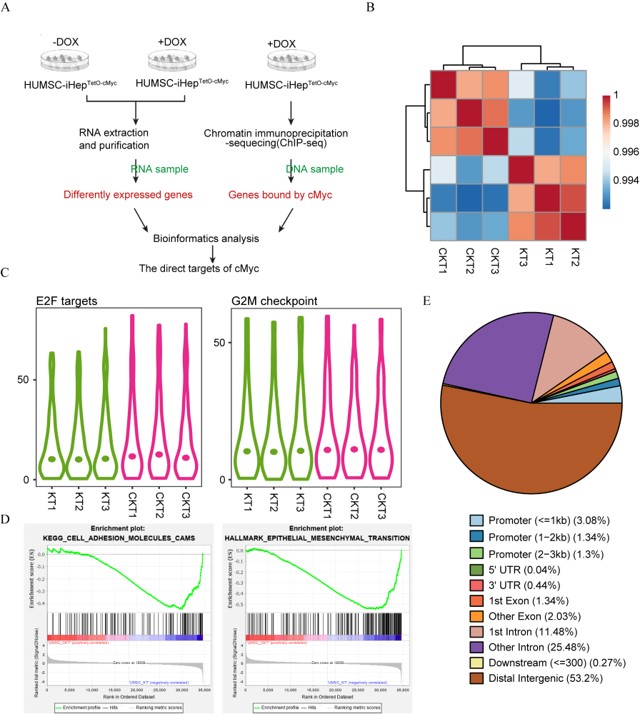


Figure S8. Regulatory network altered by c-Myc. (A) Schematic diagram depicts the experiment strategy. (B) Transcriptome correlation analysis of HUMSCs inducted by 3TFs(KT1-3) and 3TFs+c-Myc(CKT1-3). (C) Expression of genes from two gene sets: E2F targets and G2M checkpoint in two groups of HUMSCs inducted by 3TFs(KT1-3) and 3TFs+c-Myc(CKT1-3). (D) GSEA enrichment plot of two gene sets. (E) Genomic distribution of c-Myc binding sites.
